# Oncostatin M as a Complementary Non-Invasive Marker of Endoscopic and Histological Disease Activity in Ulcerative Colitis

**DOI:** 10.3390/ijms27188110

**Published:** 2026-09-11

**Authors:** Alina-Ecaterina Jucan, Carmen Atodiresei, Georgiana-Elena Sarbu, Vasile-Claudiu Mihai, Ioana-Irina Rezuș, Simona Juncu, Mariana Pavel-Tanasa, Daniela Constantinescu, Vasile Maciuc, Mihaela Dranga, Otilia Nedelciuc, Oana Barboi, Irina Ciortescu, Daniela Maria Tanase, Monica Hancianu, Vasile Drug, Cristina Cijevschi-Prelipcean, Cătălina Mihai

**Affiliations:** 1 Grigore T. Popa University of Medicine and Pharmacy Iasi, 700115 Iasi, Romania; ghiata.alina-ecaterina@d.umfiasi.ro (A.-E.J.); georgiana_ciobanu23@yahoo.com (G.-E.S.); mihai_vasile-claudiu@d.umfiasi.ro (V.-C.M.); rezus_ioana-irina@d.umfiasi.ro (I.-I.R.); juncu_simona-stefania@d.umfiasi.ro (S.J.); mariana.pavel-tanasa@umfiasi.ro (M.P.-T.); d.constantinescu@umfiasi.ro (D.C.); mihaela_dra@yahoo.com (M.D.); otilianedelciuc@yahoo.com (O.N.); oanabarboi@gmail.com (O.B.); irinaciortescu@yahoo.com (I.C.); daniela.tanase@umfiasi.com (D.M.T.); monica.hancianu@umfiasi.ro (M.H.); vasidrug@email.com (V.D.); catalina.mihai@umfiasi.ro (C.M.); 2 Department of Gastroenterology, Institute of Gastroenterology and Hepatology, “St. Spiridon” County Clinical Emergency Hospital, 700111 Iasi, Romania; cristina.cijevschi.prelipcean@umfiasi.ro; 3 Department of Radiology, “St. Spiridon” County Clinical Emergency Hospital, 700115 Iasi, Romania; 4 Department of Immunology, “St. Spiridon” County Clinical Emergency Hospital, 700111 Iasi, Romania; 5 Department of Animal Resources and Technologies, Faculty of Food and Animal Sciences, “Ion Ionescu de la Brad” University of Life Sciences, 700489 Iasi, Romania; vmaciuc@uaiasi.ro; 6 Department of Internal Medicine Clinic, “St. Spiridon” County Clinical Emergency Hospital, 700111 Iasi, Romania

**Keywords:** Oncostatin M, ulcerative colitis, histological healing, non-invasive biomarker

## Abstract

Plasma Oncostatin M (OSM) has been proposed as a biomarker of disease activity in ulcerative colitis (UC). We evaluated OSM’s relationship with clinical, endoscopic, and histological activity across two follow-up visits, compared with fecal calprotectin (FC). One hundred UC patients were assessed at Visit 1 (baseline) and Visit 2 (12 months) for clinical (partial Mayo score), endoscopic (Mayo Endoscopic Score), and histological (Nancy Histological Index) activity, alongside plasma OSM, FC, CRP, and fibrinogen; 30 healthy controls were included for comparison. Given the marked right-skewness of OSM and FC, associations were assessed using both Pearson and Spearman correlations, and logistic regression effect sizes are reported as odds ratios per doubling of biomarker concentration (log2 scale). OSM correlated significantly with all activity measures at both visits, most strongly with histological activity (r = 0.521–0.602), but weakly or not with CRP/fibrinogen. OSM was significantly elevated versus controls at both visits (*p* < 0.001) and declined significantly with treatment response (Δ = −89.06 vs. +0.50 in controls, *p* = 0.006). FC was the dominant predictor of clinical (AUC = 0.788) and endoscopic (AUC = 0.926) remission at Visit 2, with OSM contributing little independent value; however, for histological remission, both baseline OSM and ΔOSM were independent, complementary correlates (OR = 8.43 and 9.64 per doubling, respectively; *p* < 0.001 for both), yielding the strongest model overall (AUC = 0.949). OSM’s discriminative accuracy improved markedly from Visit 1 to Visit 2 for endoscopic (AUC 0.695 → 0.897) and histological (AUC 0.738 → 0.927) activity. Across two follow-up assessments, OSM consistently reflected UC activity, with its strongest and most independent signal for histological inflammation, both as a concurrent correlate and—more modestly—as a genuine prospective marker independent of baseline treatment exposure. OSM’s incremental value over FC was most apparent for histological status and for stringent (MES 0) endoscopic remission, but not for conventional clinical or endoscopic remission thresholds.

## 1. Introduction

Inflammatory bowel diseases (IBD), comprising ulcerative colitis (UC) and Crohn’s disease (CD), represent a spectrum of chronic, relapsing disorders of the gastrointestinal tract driven by a complex interplay of genetic susceptibility, epithelial barrier dysfunction, gut microbial dysbiosis, and aberrant mucosal immune activation [1,2]. Accurate prediction of UC progression and treatment response depends on understanding its dysregulated inflammatory pathways [3]. Current treatment paradigms emphasize achieving not only clinical remission but also endoscopic and, more recently, histological healing, since deeper levels of mucosal healing are associated with more durable remission and lower rates of relapse, hospitalization, and colectomy [4]. Endoscopy, the reference standard for assessing UC, is invasive, expensive, and unsuitable for repeated use, limiting its effectiveness for real-time monitoring of treatment response. Reliable non-invasive biomarkers that accurately reflect endoscopic and histological disease activity are therefore needed to reduce reliance on repeated procedures.

Current evidence identifies histological healing as an important therapeutic target, given its association with improved clinical outcomes and reduced relapse rates [5]. Although fecal calprotectin (FC) is the most widely used non-invasive biomarker for UC monitoring, its limitations in accurately detecting mild residual inflammation and histological healing have driven the search for complementary serum biomarkers with greater pathophysiological relevance [6,7].

Oncostatin M (OSM) is a pro-inflammatory cytokine of the IL-6 family, produced by activated T cells, monocytes, macrophages, dendritic cells, and neutrophils, and signaling through the shared gp130 receptor subunit [8,9]. OSM and its receptor (OSMR) are overexpressed in inflamed intestinal tissue in IBD, and prior work has shown that OSM expression correlates with histological disease severity and is associated with resistance to anti-TNF therapy, suggesting a mechanistic link between OSM-driven stromal and immune cell signaling and treatment-refractory inflammation [9,10]. Evidence from subsequent studies indicates that OSM levels correlate with disease activity and, in some patient cohorts, with mucosal healing. Owing to its treatment-specific dynamics and distinct position in the inflammatory signaling relative to FC and CRP, OSM may represent a valuable complementary biomarker for monitoring UC [11].

Despite growing evidence supporting the biological role of OSM as a biomarker, longitudinal studies directly comparing its performance with FC across clinical, endoscopic, and histological outcomes within the same patient cohort remain limited. Clarifying whether OSM offers incremental value over FC or reflects a comparable inflammatory signal is crucial for its integration into treat-to-target approaches in UC. Accordingly, this study evaluated OSM levels in patients with UC compared with healthy controls, investigated their longitudinal relationship with disease activity using clinical, endoscopic, and histological indices, and assessed whether OSM adds diagnostic value beyond FC for remission.

## 2. Results

### 2.1. Study Population

A descriptive analysis, illustrated in Table 1, was performed for all variables. A total of 100 patients with UC were included in the study. The mean age was 41.96 ± 14.39 years, and there was a predominance of males (62%). The mean BMI was 25.24 ± 4.21 kg/m^2^, corresponding overall to the overweight range. Active smoking and alcohol consumption were reported in 10% and 2% of patients, respectively, while extraintestinal manifestations were present in 29% of cases.

Regarding disease phenotype, pancolitis (Montreal E3) represented the most frequent disease extent, affecting 55% of patients, followed by left-sided colitis (39%) and proctitis (6%). At baseline, the majority of patients presented with moderate-to-severe disease activity. Clinical and endoscopic activity scores improved during follow-up, with the pMS decreasing from 4.07 ± 1.82 at Visit 1 to 3.28 ± 2.21 at Visit 2, and the MES decreasing from 1.86 ± 0.84 to 1.40 ± 0.98. Histological activity assessed by the Nancy score also showed a slight reduction over time.

A significant shift toward lower disease severity was observed during follow-up. According to the Truelove and Witts classification, the proportion of patients with mild disease increased from 31% to 58%, whereas severe disease decreased from 22% to 8%. In parallel, inflammatory biomarkers demonstrated a consistent downward trend. Mean FC levels decreased from 692.77 ± 1162.38 to 417.97 ± 637.72, while OSM levels declined from 222.36 ± 398.43 to 133.30 ± 242.48. Similarly, fibrinogen and CRP values were lower at the second evaluation, suggesting an overall reduction in systemic and intestinal inflammatory burden.

### 2.2. OSM Changes Between Visits

Changes in OSM levels were moderately correlated with changes in clinical, endoscopic, histological, and fecal inflammatory markers, suggesting that OSM reflects variations in intestinal disease activity over time. In contrast, no significant correlations were observed with CRP or fibrinogen, indicating that OSM may better reflect local intestinal inflammation than systemic inflammatory response markers. Furthermore, higher baseline disease activity was associated with greater reductions in OSM, supporting its potential role as a dynamic biomarker of treatment response.

#### 2.2.1. OSM Visit 1 Correlations

**Figure 1:**
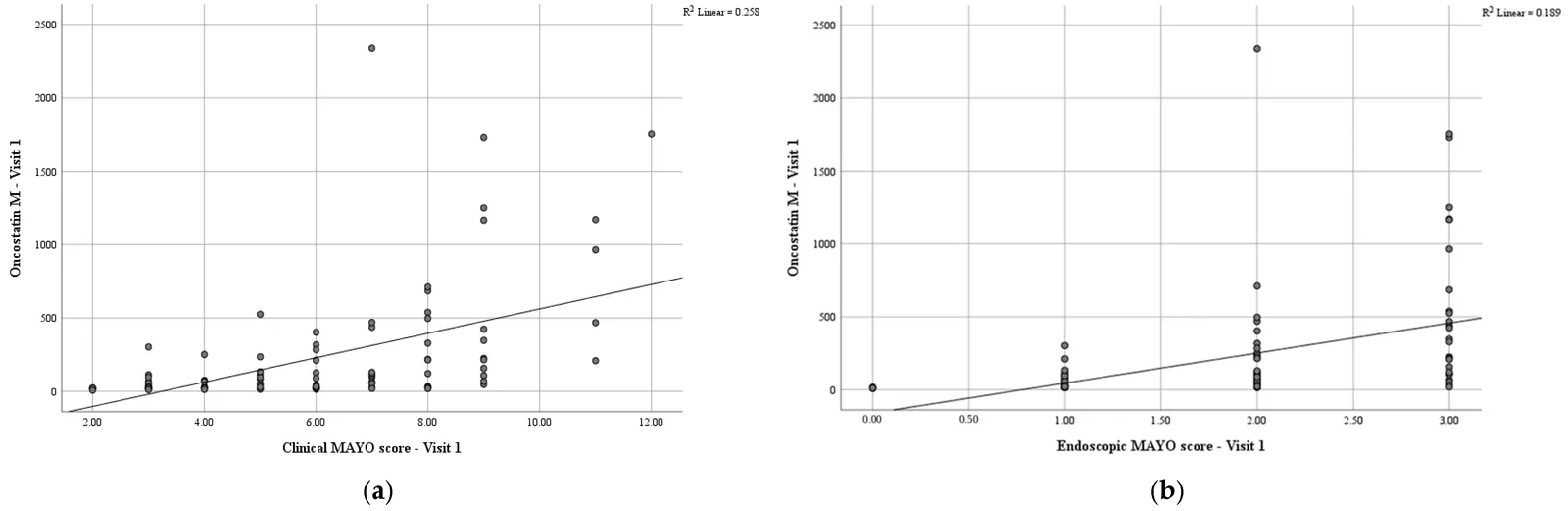

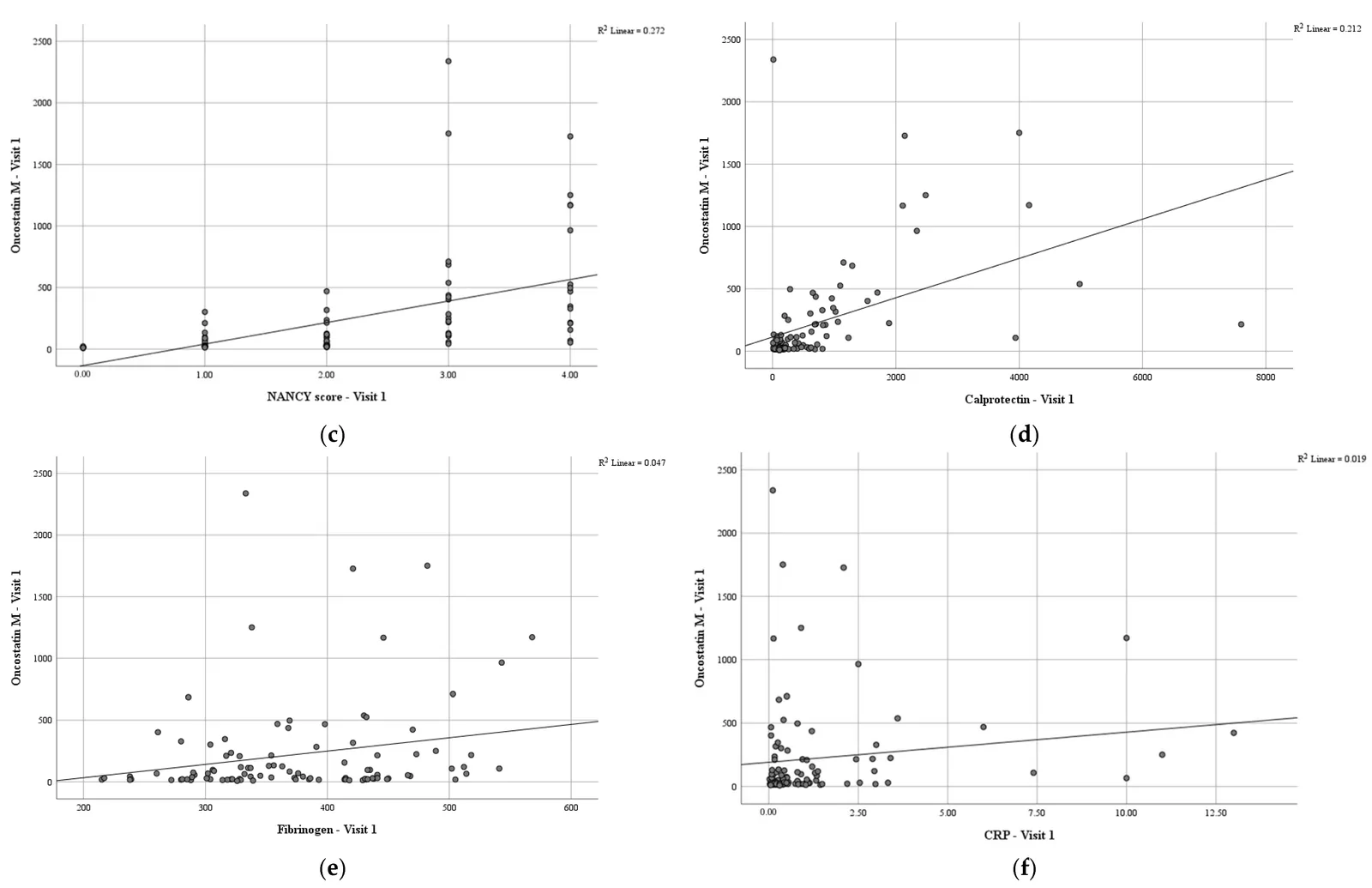
Scatterplots showing Pearson correlations between plasma OSM levels and disease activity/inflammatory markers in patients with UC (*n* = 100). Each panel displays a linear regression line with 95% confidence interval. (**a**) pMS (r = 0.508, *p* < 0.001); (**b**) MES (r = 0.435, *p* < 0.001); (**c**) NHI (r = 0.521, *p* < 0.001); (**d**) FC (r = 0.460, *p* < 0.001); (**e**) Fibrinogen (r = 0.217, *p* = 0.030); (**f**) CRP (r = 0.139, *p* = 0.167, not statistically significant). Correlations were significant for all markers except CRP, ranging from weak (fibrinogen) to moderate (pMS, NHI).

Considering that pMS, CRP, and fibrinogen are continuous variables, Pearson correlation coefficients were calculated to assess their linear relationships with OSM levels. The results of our analysis showed that there was a significant moderate positive Pearson correlation between OSM levels and pMS, r(98) = 0.508, *p* < 0.001, indicating that higher OSM levels were associated with increased disease activity—Figure 1a. A significant moderate positive Pearson correlation was observed between OSM levels and the endoscopic Mayo score, r(98) = 0.435, *p* < 0.001, suggesting that higher OSM values were associated with more severe endoscopic inflammation—Figure 1b. OSM levels showed a significant moderate to strong positive Pearson correlation with the Nancy score, r(98) = 0.521, *p* < 0.001, indicating a robust association between OSM and histological disease activity—Figure 1c. The Pearson correlation results demonstrated a significant moderate positive association between OSM and FC, r(98) = 0.460, *p* < 0.001, reflecting the relationship between OSM and biochemical markers of intestinal inflammation—Figure 1d. The correlation between OSM and fibrinogen was statistically significant but weak, r(98) = 0.217, *p* = 0.030, suggesting only a small linear association between the two variables—Figure 1e. OSM levels showed a non-significant Pearson correlation with CRP, r(98) = 0.139, *p* = 0.167, indicating no meaningful linear relationship—Figure 1f.

#### 2.2.2. OSM Visit 2 Correlations

**Figure 2:**
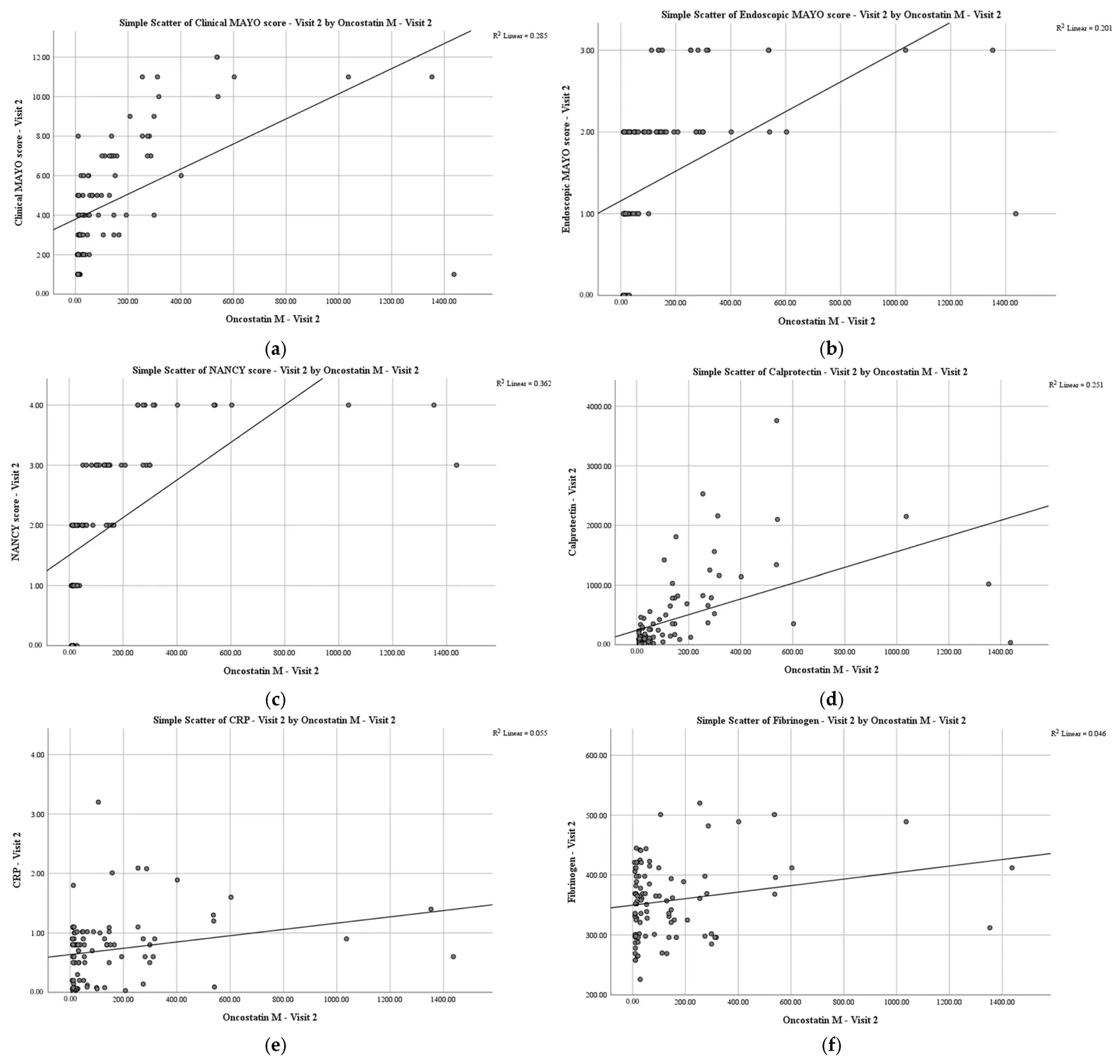
Scatterplots showing Pearson correlations between plasma OSM levels and disease activity/inflammatory markers at Visit 2 (*n* = 100). Each panel displays a linear regression line with 95% confidence interval. (**a**) pMS (r = 0.534, *p* < 0.001); (**b**) MES (r = 0.448, *p* < 0.001); (**c**) Nancy histological score (r = 0.602, *p* < 0.001); (**d**) FC (r = 0.501, *p* < 0.001); (**e**) CRP (r = 0.236, *p* = 0.018); (**f**) Fibrinogen (r = 0.214, *p* = 0.032). All correlations were statistically significant, ranging from weak (CRP, fibrinogen) to strong (Nancy score).

Pearson correlation analysis demonstrated significant associations between plasma OSM and all evaluated activity scores at Visit 2. OSM showed a moderate positive correlation with the pMS (r = 0.534, *p* < 0.001)—Figure 2a and with the MES (r = 0.448, *p* < 0.001)—Figure 2b. A strong positive correlation was observed between OSM and the NANCY histological score (r = 0.602, *p* < 0.001)—Figure 2c. OSM was also moderately correlated with FC (r = 0.501, *p* < 0.001)—Figure 2d. Weak but statistically significant correlations were identified with CRP (r = 0.236, *p* = 0.018)—Figure 2e and fibrinogen (r = 0.214, *p* = 0.032)—Figure 2f.

Given the marked right-skewness of OSM and FC (skewness 2.6–3.6; Shapiro–Wilk *p* < 0.001), Spearman’s rank correlation coefficients were also calculated. Spearman correlations were consistently at least as strong as, and in most cases stronger than, the corresponding Pearson coefficients (OSM vs. pMS: r = 0.508/ρ = 0.598 at V1, r = 0.534/ρ = 0.700 at V2; OSM vs. MES: r = 0.435/ρ = 0.615 at V1, r = 0.448/ρ = 0.753 at V2; OSM vs. NHI: r = 0.521/ρ = 0.738 at V1, r = 0.602/ρ = 0.861 at V2; OSM vs. FC: r = 0.460/ρ = 0.589 at V1, r = 0.501/ρ = 0.669 at V2).

#### 2.2.3. Delta Associations

Pearson correlation analysis demonstrated significant associations between ΔOSM and the evaluated Δmarkers and Δactivity scores. ΔOSM showed a moderate positive correlation with ΔCalprotectin (r = 0.446, *p* < 0.001) and with ΔClinical MAYO score (r = 0.452, *p* < 0.001). Similar moderate correlations were observed with ΔMES (r = 0.446, *p* < 0.001) and ΔNANCY (r = 0.461, *p* < 0.001). No significant correlations were identified between ΔOSM and ΔCRP (r = 0.096, *p* = 0.343) or ΔFibrinogen (r = 0.127, *p* = 0.208). The correlation between ΔOncostatin M and age was weak and non-significant, r(98) = 0.160, *p* = 0.113, indicating no age-related pattern in OSM. Also, ΔOncostatin M showed a weak and non-significant Pearson correlation with BMI, r(98) = 0.133, *p* = 0.188, indicating no association between body composition and OSM variation. In contrast, ΔOncostatin M showed a statistically significant weak negative Spearman correlation with disease extent, ρ = −0.226, *p* = 0.024, suggesting that patients with more extensive disease tended to exhibit smaller increases—or greater decreases—in OSM levels over time—Table 2.

Moreover, a multiple linear regression analysis was performed to predict ΔOSM based on ΔCalprotectin, ΔNANCY, and Nancy remission status at Visit 2. The overall model was statistically significant, F(3, 96) = 14.80, *p* < 0.001, and explained 31.6% of the variance in ΔOSM (R^2^ = 0.316; R = 0.562, indicating a moderate-to-large combined association). ΔNANCY was the strongest positive predictor (β = 0.441, *p* < 0.001), followed by ΔCalprotectin (β = 0.251, *p* = 0.011). Achieving Nancy histologic remission at Visit 2 was a significant negative predictor (β = −0.214, *p* = 0.029), indicating that patients who reached remission showed smaller increases (or greater decreases) in OSM compared to those who did not. Multicollinearity was not a concern, as Variance Inflation Factor (VIF) values remained low (ΔCalprotectin = 1.331, ΔNANCY = 1.667, Nancy remission = 1.310).

#### 2.2.4. Repeated-Measures (Mixed-Effects) Analysis

To account for the correlated, within-patient structure of the two-visit design, we fitted random-intercept linear mixed-effects models with log-transformed OSM as the fixed-effect predictor, patient as a random intercept, and visit included as a covariate, pooling both visits (*n* = 200 observations, 100 patients). Log(OSM) remained a highly significant predictor of pMS (β = 1.34, SE = 0.11, *p* < 0.0001), MES (β = 0.46, SE = 0.04, *p* < 0.0001), and NHI (β = 0.72, SE = 0.04, *p* < 0.0001). Intraclass correlation coefficients (0.23–0.32) confirmed meaningful within-patient correlation across visits.

### 2.3. Association of OSM with Remission Status at Visit 2

In the analyses that follow, we distinguish three related but analytically distinct questions: (i) whether baseline biomarker values, together with subsequent change, are associated with disease status at Visit 2 (Section 2.3.1, Section 2.3.2, Section 2.3.3 and Section 2.3.4), interpreted as concurrent diagnostic association since a model containing both baseline OSM and ΔOSM together mathematically reconstructs the Visit 2 OSM value; (ii) whether baseline biomarkers alone, without any Visit 2 information, prospectively predict Visit 2 histological status (Section 2.3.5); and (iii) whether biomarker levels measured at Visit 2 discriminate active from inactive disease measured at the same visit (Section 2.5). We reserve “predict”/“predictor” exclusively for the genuinely prospective analysis in Section 2.3.5.

To evaluate the concurrent association of changes in inflammatory markers (ΔOSM and ΔCalprotectin) on the clinical status of active disease at Visit 2, a binary logistic regression model adjusted for baseline values (Visit 1) was conducted.

#### 2.3.1. Association of Baseline and Change Values with Clinical Status at Visit 2

**Figure 3:**
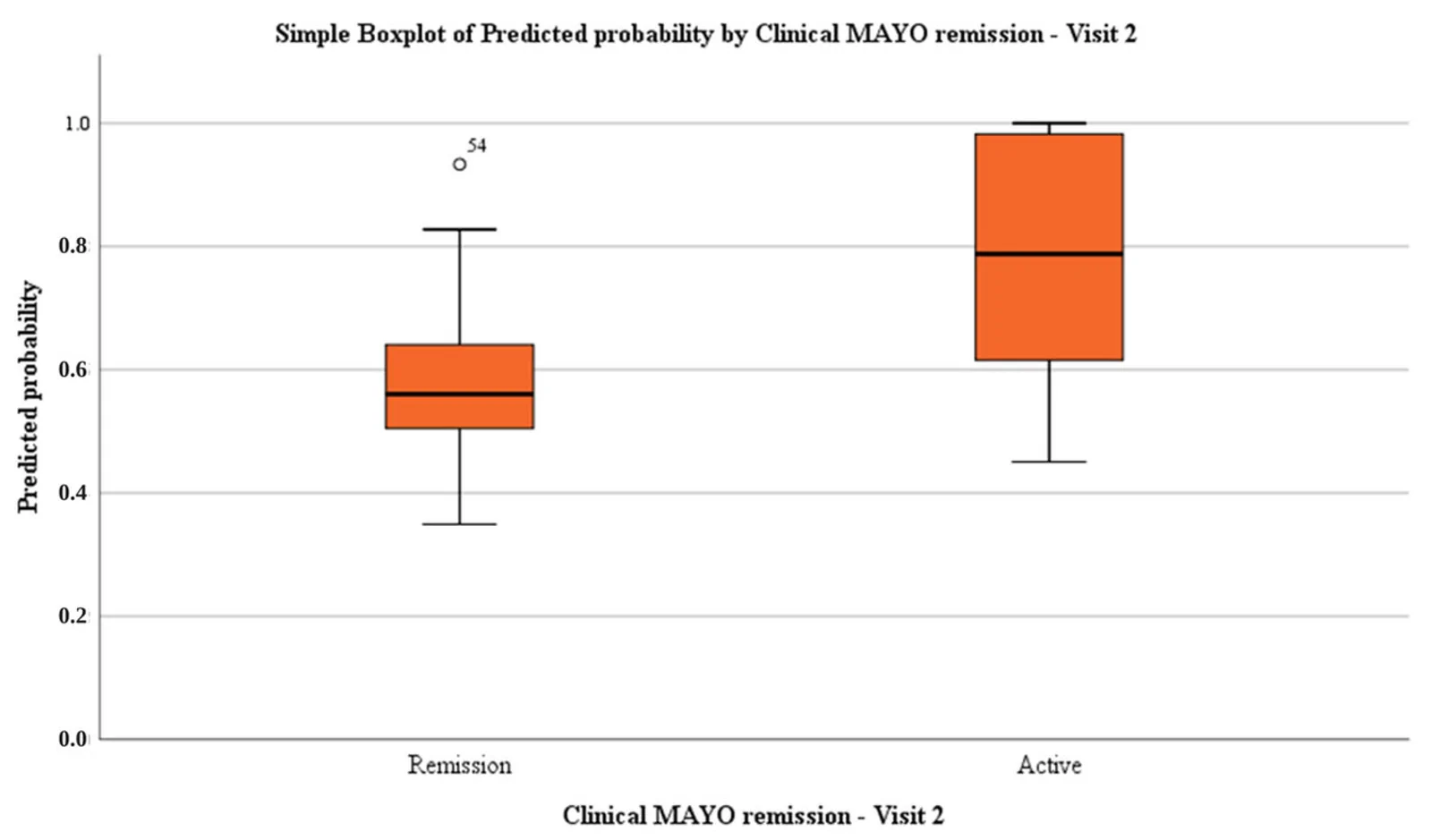
Distribution of predicted probabilities from the logistic regression model. The boxplot shows how effectively the model separates patients in clinical remission from those with active clinical disease at Visit 2 based on baseline and delta values of FC and OSM. Circle (○)—labeled 54—is a mild outlier.

The Omnibus test showed that the overall model is highly significant, Chi-square(4) = 24.236, *p* < 0.001, meaning this combination of variables works well to classify clinical status at Visit 2. The model has a strong explanatory power (−2 Log likelihood = 92.415) and explains between 21.5% (Cox & Snell R Square) and 31.3% (Nagelkerke R Square) of the variance in the final clinical status—Figure 3.

Looking at the individual predictors using the clinical score, baseline FC is significantly associated with active clinical disease at Visit 2 (B = 0.362, SE = 0.172, *p* = 0.035, OR = 1.44). ΔFC does not reach significance in this corrected analysis (B = 0.274, SE = 0.168, *p* = 0.104, OR = 1.32). Baseline OSM (B = 0.318, SE = 0.172, *p* = 0.065, OR = 1.38) and ΔOSM (B = 0.354, SE = 0.199, *p* = 0.075, OR = 1.43) approach but do not reach conventional significance. The model’s discriminative accuracy was AUC = 0.779 (95% CI, *n* = 99).

In addition, to ensure the statistical validity of the model, collinearity diagnostics were conducted for all independent variables. The Variance Inflation Factor (VIF) values confirm that multicollinearity did not adversely impact the stability of the regression coefficients: OSM (Visit 1) VIF = 3.630, FC (Visit 1) VIF = 4.707, Δ OSM VIF = 3.526, and Δ Calprotectin VIF = 4.625.

#### 2.3.2. Association of Baseline and Change Values with Endoscopic Status at Visit 2

**Figure 4:**
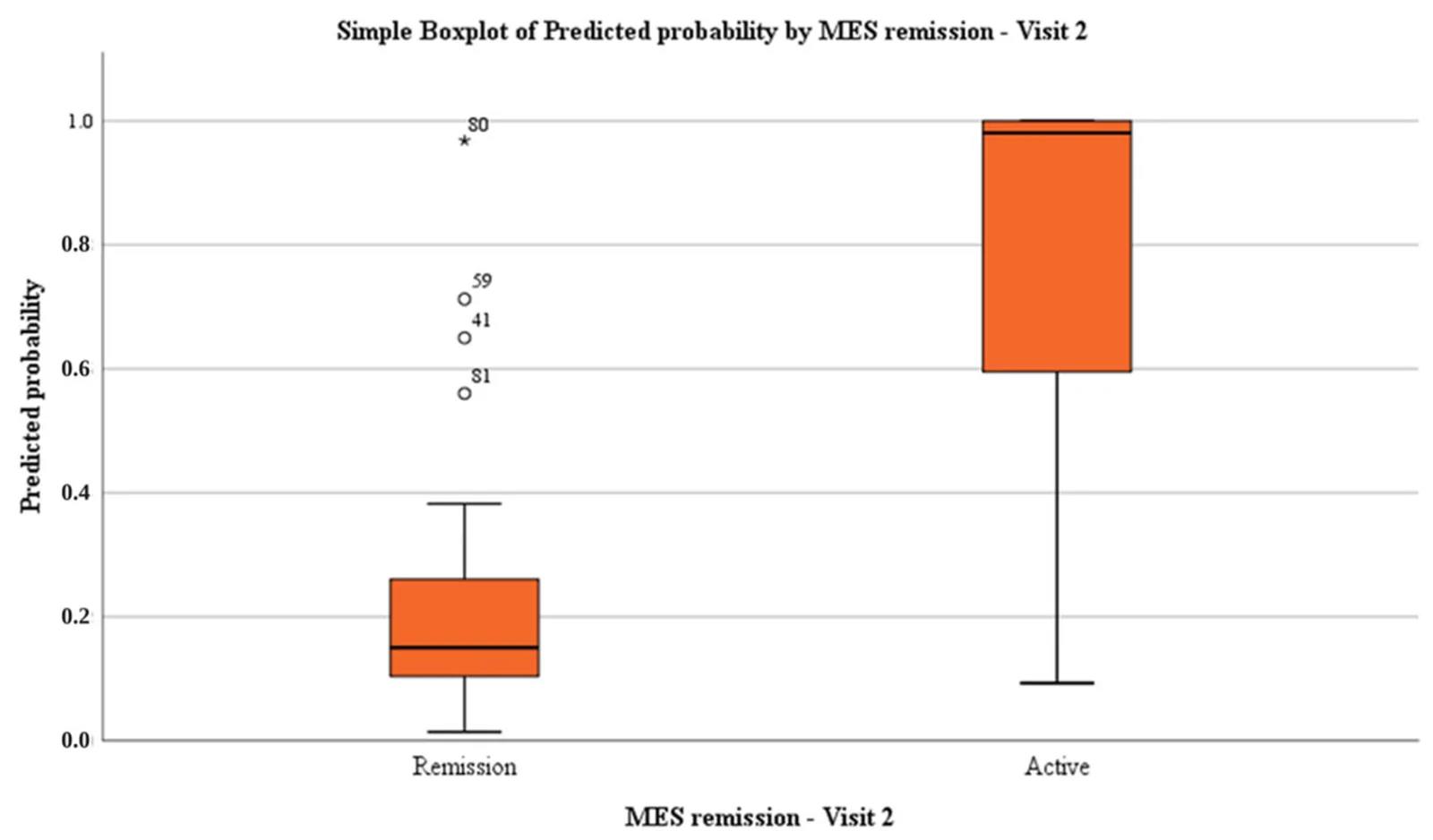
Distribution of predicted probabilities from the logistic regression model. The boxplot shows how effectively the model separates patients in endoscopic remission from those with active endoscopic disease at Visit 2 based on baseline and delta values of FC and OSM. Circles (○)—labeled 41, 59, 81—are mild outliers. Asterisk (*)—labeled 80—is an extreme outlier.

The Omnibus test showed that the overall model is highly significant, Chi-square(4) = 71.013, *p* < 0.001, meaning this combination of variables works well to classify endoscopic status at Visit 2. The model has a strong explanatory power (−2 Log likelihood = 67.616) and explains between 50.8% (Cox & Snell R Square) and 67.8% (Nagelkerke R Square) of the variance in the final endoscopic status—Figure 4.

Looking at the individual predictors, both the baseline level and the change in FC are strong, independent correlates of active disease at Visit 2. ΔCalprotectin is highly significant (B = 0.012, SE = 0.003, Wald = 15.065, *p* < 0.001, OR = 1.012, 95% CI [1.006, 1.019]), which means that for every 1-unit increase in the delta value (meaning a smaller drop or a larger increase in inflammation between visits), the odds of having active endoscopic disease at Visit 2 increase by 1.2%. FC at Visit 1 is also highly significant (B = 0.013, SE = 0.003, Wald = 15.949, *p* < 0.001, OR = 1.013, 95% CI [1.007, 1.019]), showing that patients who start with a higher inflammatory burden at baseline have 1.3% higher odds of keeping an active disease at the second visit, regardless of the delta change.

On the other hand, OSM parameters do not add to the concurrent association when FC is in the model. ΔOSM showed only a weak, non-significant trend (B = 0.005, SE = 0.003, Wald = 3.077, *p* = 0.079, OR = 1.005, 95% CI [0.999, 1.011]), and baseline OSM at Visit 1 had no statistical impact at all (B = 0.003, SE = 0.002, Wald = 2.197, *p* = 0.138, OR = 1.003, 95% CI [0.999, 1.006]). In short, when looking at endoscopic remission outcomes, FC is the dominant correlate and overrides the association of OSM.

The global diagnostic accuracy of the model is demonstrated by an excellent discriminative power, with an AUC of 0.926 (95% CI [0.875, 0.977], *p* < 0.001), as visually represented in the ROC Curve.

#### 2.3.3. Association of Baseline and Change Values with Histological Status at Visit 2

**Figure 5:**
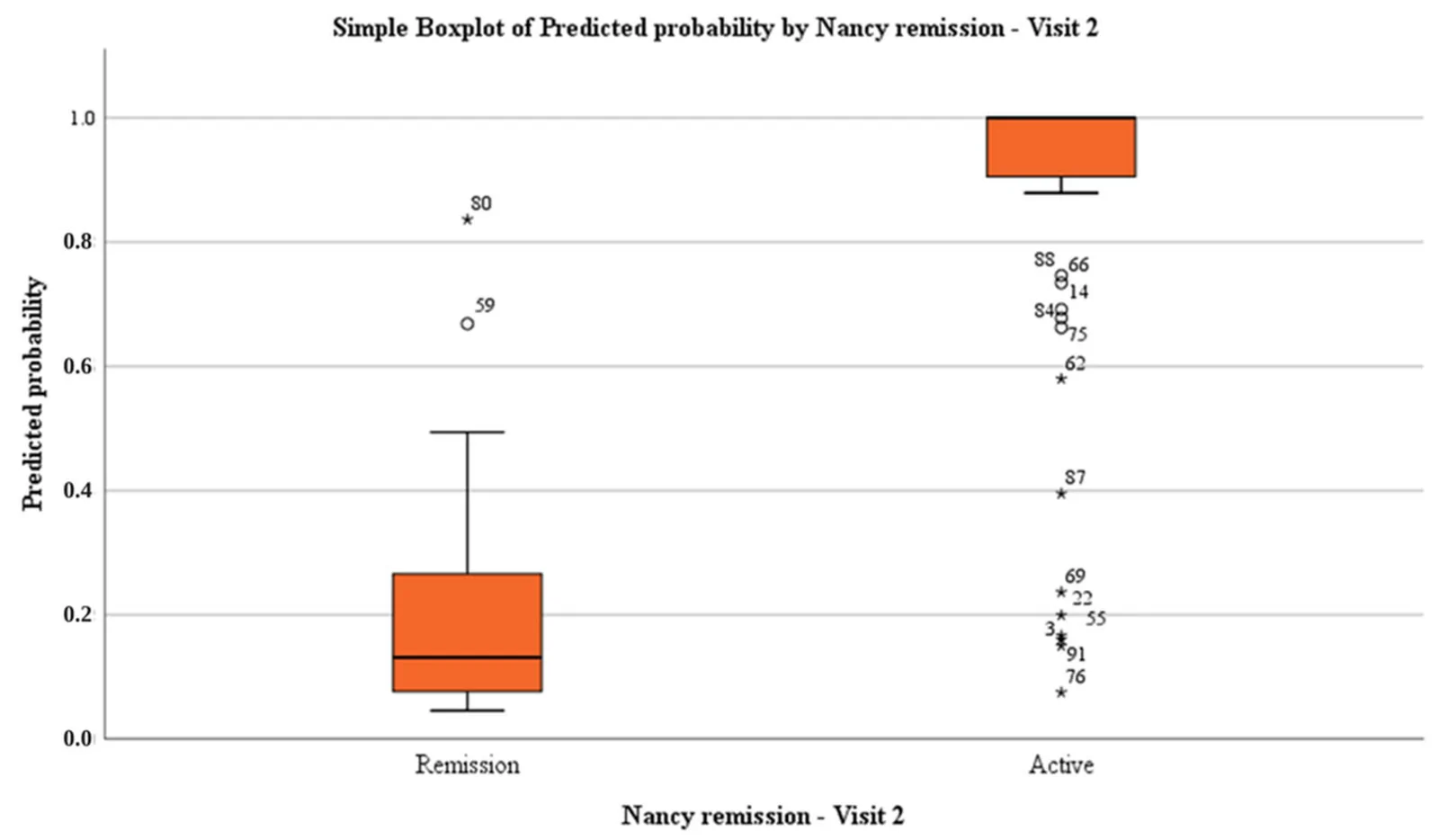
Distribution of predicted probabilities from the logistic regression model. The boxplot shows how effectively the model separates patients in histological remission from those with active histological disease at Visit 2 based on baseline and delta values of FC and OSM. Circles (○)—labeled 66, 14, 84, 75, 88, 59—are mild outliers. Asterisks (*)—labeled 80, 62, 87, 69, 22, 55, 91, 76, 3—are extreme outliers.

The Omnibus test showed that the overall model is highly significant, Chi-square(4) = 83.538, *p* < 0.001, meaning this combination of variables works well to classify histological status at Visit 2. The model has a strong explanatory power (−2 Log likelihood = 51.064) and explains between 56.6% (Cox & Snell R Square) and 76.6% (Nagelkerke R Square) of the variance in the final histological status—Figure 5.

Looking at the individual predictors, both the baseline level and the change in FC are strong, independent predictors of active disease at Visit 2. ΔCalprotectin is highly significant (B = 0.009, SE = 0.004, Wald = 5.584, *p* = 0.018, OR = 1.009, 95% CI [1.001, 1.016]), which means that for every 1-unit increase in the delta value (meaning a smaller drop or a larger increase in inflammation between visits), the odds of having active histological disease at Visit 2 increase by 0.9%. FC at Visit 1 is also highly significant (B = 0.009, SE = 0.004, Wald = 5.691, *p* = 0.017, OR = 1.009, 95% CI [1.002, 1.016]), showing that patients who start with a higher inflammatory burden at baseline have 0.9% higher odds of having an active disease at the second visit, regardless of the delta change.

On the other hand, OSM parameters also emerge as highly significant, independent predictors in this model. ΔOSM showed a strong, significant effect (B = 0.105, SE = 0.031, Wald = 11.386, *p* = 0.001, OR = 1.111, 95% CI [1.045, 1.180]), indicating that for every 1-unit increase in the delta value, the odds of active histological disease increase by 11.1%. Similarly, baseline OSM at Visit 1 had a highly significant statistical impact (B = 0.105, SE = 0.031, Wald = 11.559, *p* = 0.001, OR = 1.111, 95% CI [1.045, 1.180]), increasing the odds by 11.1% per unit. In short, when looking at concurrent histological status, FC does not override the association of OSM; instead, both markers are independently and complementarily associated with tissue-level clearance. Because ΔOSM = OSM (Visit 2) − OSM(Visit 1), a model containing both baseline OSM and ΔOSM together mathematically reconstructs OSM at Visit 2; this analysis therefore characterizes concurrent diagnostic association. Although baseline OSM and ΔOSM appear numerically identical at three decimal places, higher-precision estimates confirm they are distinct coefficients (baseline OSM: B = 0.1050, SE = 0.0309, Wald = 11.559; ΔOSM: B = 0.1048, SE = 0.0311, Wald = 11.386). Their close similarity reflects the collinearity between baseline OSM and its own change score noted above (VIF ≈ 3.6 for both terms), consistent with the model distributing a shared effect on Visit 2 OSM levels nearly evenly across the two correlated terms.

The global diagnostic accuracy of the model is demonstrated by an outstanding discriminative power, with an AUC of 0.949 (95% CI [0.909, 0.989], *p* < 0.001), as visually represented in the ROC Curve. Because OSM and FC were markedly right-skewed, we additionally report effect estimates on a log2 scale (odds ratio per doubling), since raw per-pg/mL odds ratios are not clinically interpretable given the several-hundred-pg/mL range of these markers. On this scale, baseline OSM (OR = 8.43 per doubling, 95% CI [2.68, 26.53], *p* < 0.001) and ΔOSM (OR = 9.64 per doubling, 95% CI [2.84, 32.69], *p* < 0.001) remained highly significant, as did baseline FC (OR = 3.97 per doubling, *p* = 0.003) and ΔFC (OR = 2.39 per doubling, *p* = 0.014).

#### 2.3.4. Incremental Value of OSM over FC (Nested Model Comparison)

To directly address whether OSM provides incremental diagnostic value beyond FC—the central clinical question motivating this study—we compared, for each Visit 2 outcome, a baseline model containing only FC parameters (FC at Visit 1 and ΔCalprotectin) against a nested model additionally including OSM parameters (OSM at Visit 1 and ΔOSM). Model fit was compared using a likelihood ratio test (df = 2), and discriminative performance was compared using DeLong’s test for correlated ROC curves fitted on the same patients.

For clinical remission, the addition of OSM to the FC-only model did not significantly improve model fit (likelihood ratio χ^2^(2) = 0.69, *p* = 0.710) and did not significantly change the AUC (FC alone: 0.793, 95% CI; FC + OSM: 0.788; DeLong Z = 0.38, *p* = 0.707). For endoscopic remission, the addition of OSM likewise did not significantly improve model fit (χ^2^(2) = 3.51, *p* = 0.173) or AUC (FC alone: 0.936; FC + OSM: 0.926; DeLong Z = 0.88, *p* = 0.377). In both cases, the point estimate for the combined model’s AUC was numerically slightly lower than the FC-alone model, consistent with OSM adding negligible or slightly redundant information once FC is accounted for.

In contrast, for histological remission, adding OSM to the FC-only model produced a highly significant improvement in model fit (χ^2^(2) = 35.35, *p* < 0.001) and a significant increase in discriminative accuracy (FC alone: 0.882; FC + OSM: 0.949; DeLong Z = −2.14, *p* = 0.033)—Table 3.

Taken together, these formally tested, nested model comparisons confirm that OSM does not provide meaningful incremental value over FC for detecting clinical or endoscopic remission, but does provide substantial, statistically significant incremental value for detecting histological remission—reinforcing OSM’s specific role as a complementary marker of tissue-level, rather than clinical or endoscopic, disease activity.

At the Youden-optimal cutoff, the combined model achieved sensitivity of 0.850, specificity of 0.975, PPV of 0.981, and NPV of 0.812 for histological remission. The combined model showed good calibration (Hosmer–Lemeshow χ^2^ = 1.36, df = 8, *p* = 0.995). Bootstrap internal validation (500 resamples) gave optimism-corrected AUC estimates of 0.880 (FC alone) and 0.953 (FC + OSM), close to the apparent estimates (0.886 and 0.964), suggesting limited overfitting.

#### 2.3.5. Baseline-Only Prospective Prediction of Histological Remission

To provide a genuinely prospective test of whether baseline OSM predicts subsequent histological status—using only data available at Visit 1, without incorporating any Visit 2 data—we fitted a baseline-only logistic regression model (baseline FC and baseline OSM only, no change scores) predicting histological remission at Visit 2. Baseline FC alone showed modest discriminative accuracy (AUC = 0.679). Adding baseline OSM significantly improved the model (likelihood ratio χ^2^(1) = 8.81, *p* = 0.003), increasing the AUC to 0.749, with baseline OSM independently associated with subsequent histological status (OR = 1.51 per doubling, 95% CI [1.12, 2.03], *p* = 0.007, after adjustment for baseline advanced-therapy exposure (likelihood ratio χ^2^(1) = 8.61, *p* = 0.003)). This baseline-only analysis constitutes the manuscript’s genuinely prospective evidence that OSM adds predictive value over FC; the larger effect sizes reported in Section 2.3.3 and Section 2.3.4, which additionally incorporate ΔOSM, should be interpreted as reflecting concurrent diagnostic association with Visit 2 disease status rather than prospective prediction, as ΔOSM together with baseline OSM mathematically reconstructs the Visit 2 OSM value.

#### 2.3.6. Treatment Exposure and Sensitivity Analysis

Treatment distributions at Visit 1 and Visit 2 are shown in Table 4. Advanced therapy (biologic or small molecule) use increased from 62% at Visit 1 to 83% at Visit 2, corticosteroid use decreased from 24% to 2%, and 41 of 100 patients had a change in treatment category between visits, including 21 patients who escalated to advanced therapy during follow-up. To assess whether treatment change confounded the association between OSM and histological remission, we added treatment escalation (yes/no) as a covariate to the primary FC + OSM histological model (Section 2.3.3). Baseline OSM (OR = 8.84, *p* < 0.001) and ΔOSM (OR = 9.91, *p* < 0.001) remained highly significant and materially unchanged in magnitude after adjustment, while treatment escalation itself was not significantly associated with histological outcome (OR = 2.96, *p* = 0.41), indicating that the OSM–histological remission association is not substantially explained by treatment escalation during the study period.

### 2.4. Differences in OSM Levels Between Study and Control Groups

Assumption testing. Levene’s test indicated unequal variances for all comparisons (Visit 1: F = 17.61, *p* < 0.001; Visit 2: F = 14.88, *p* < 0.001; ΔOSM: F = 14.23, *p* < 0.001), therefore Welch’s *t*-test results were used for interpretation.

**Figure 6:**
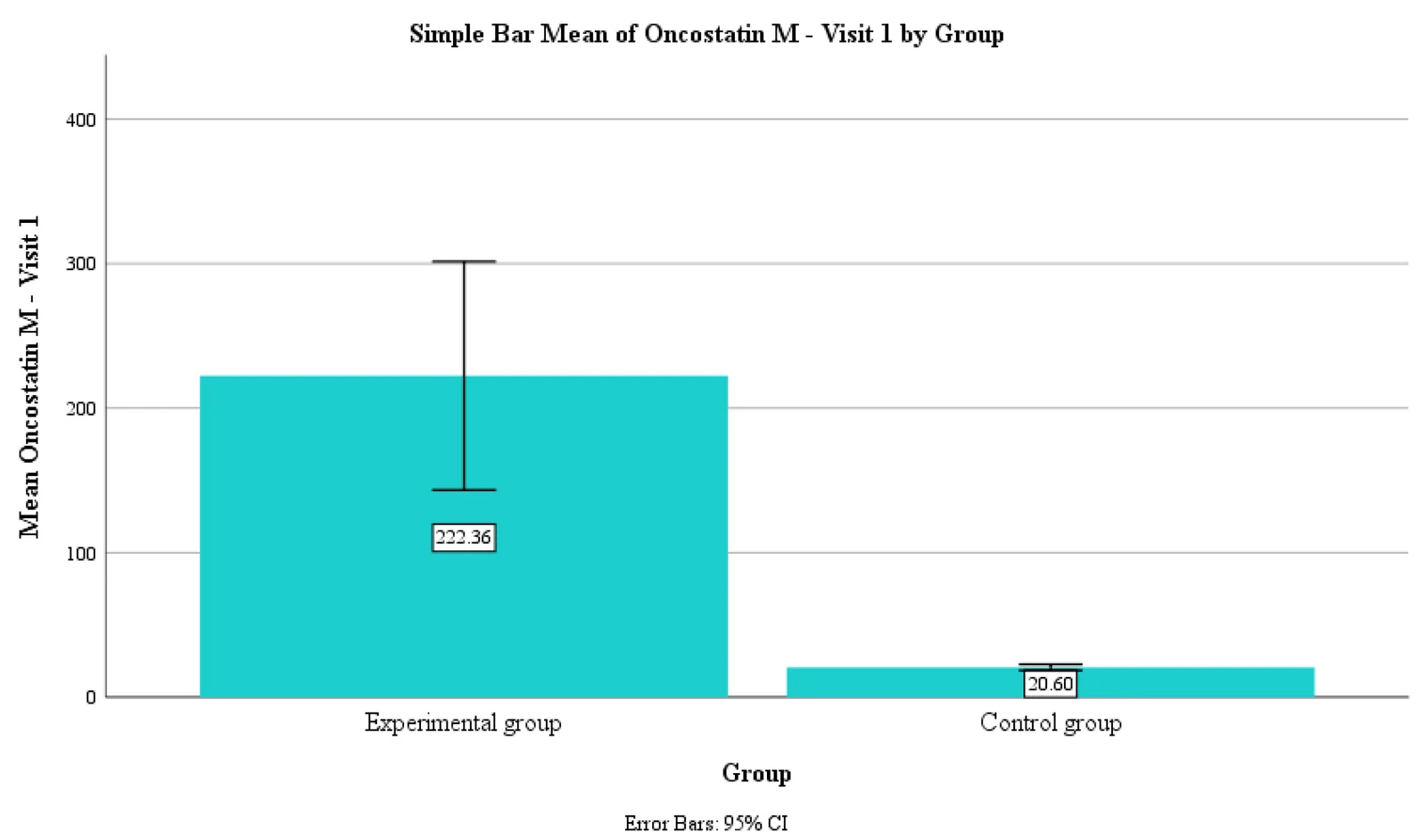
OSM levels at Visit 1 in the study and control groups. Data are presented as mean ± 95% confidence interval.

OSM levels at Visit 1 and Visit 2. An independent-samples *t*-test (Welch’s correction) showed that OSM levels at Visit 1 were significantly higher in the study group (M = 222.36, SD = 398.43, SE = 39.84, *n* = 100) compared with the control group (M = 20.60, SD = 5.69, SE = 1.04, *n* = 30), t(99.13) = 5.06, *p* < 0.001, mean difference = 201.76, 95% CI [122.68, 280.85]—Figure 6.

**Figure 7:**
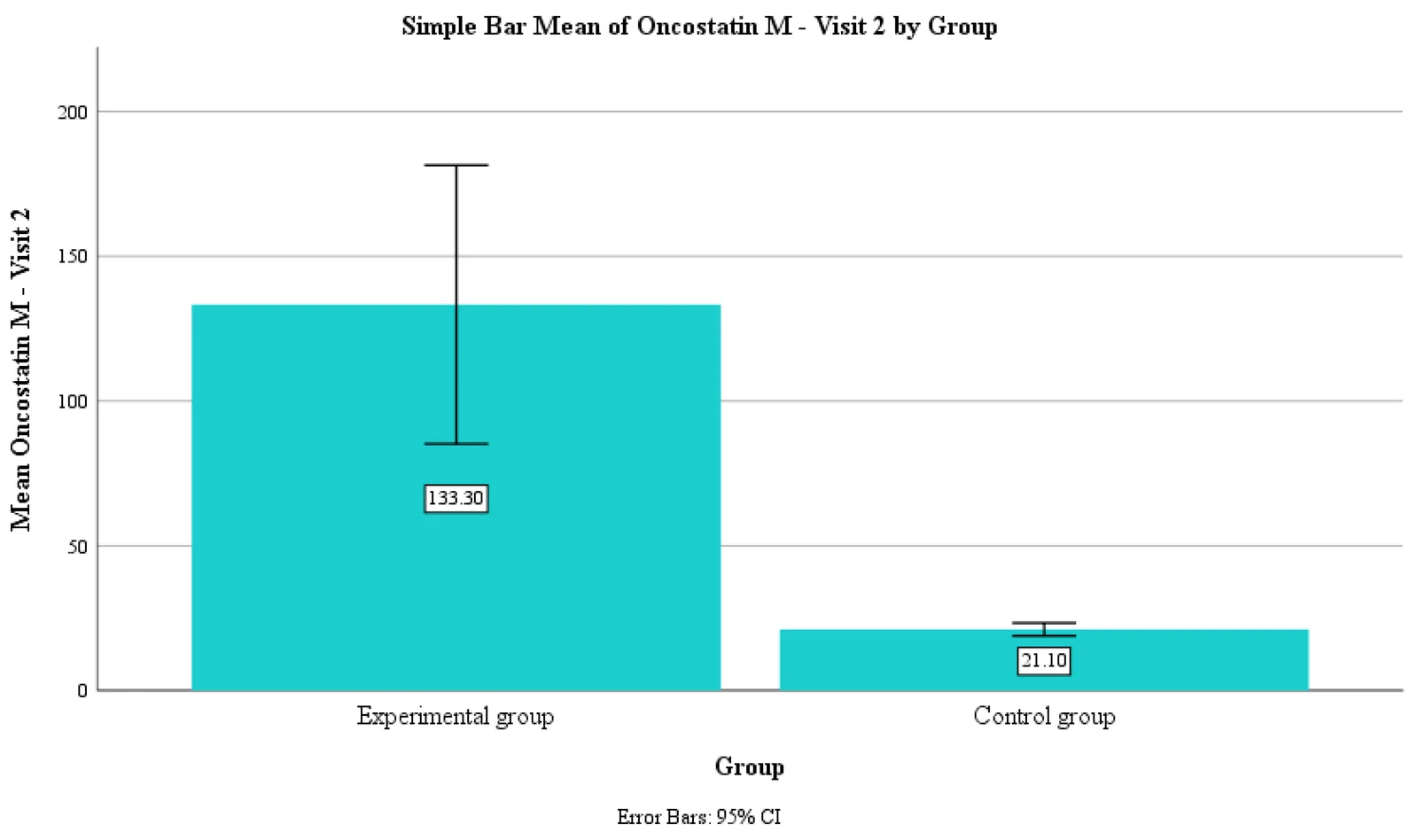
OSM levels at Visit 2 in the study and control groups. Data are presented as mean ± 95% confidence interval.

At Visit 2, OSM levels remained significantly higher in the study group (M = 133.30, SD = 242.48, SE = 24.25, *n* = 100) than in the control group (M = 21.10, SD = 5.95, SE = 1.09, *n* = 30), t(99.40) = 4.62, *p* < 0.001, mean difference = 112.20, 95% CI [64.04, 160.36]—Figure 7.

#### Change in OSM (ΔOSM)

**Figure 8:**
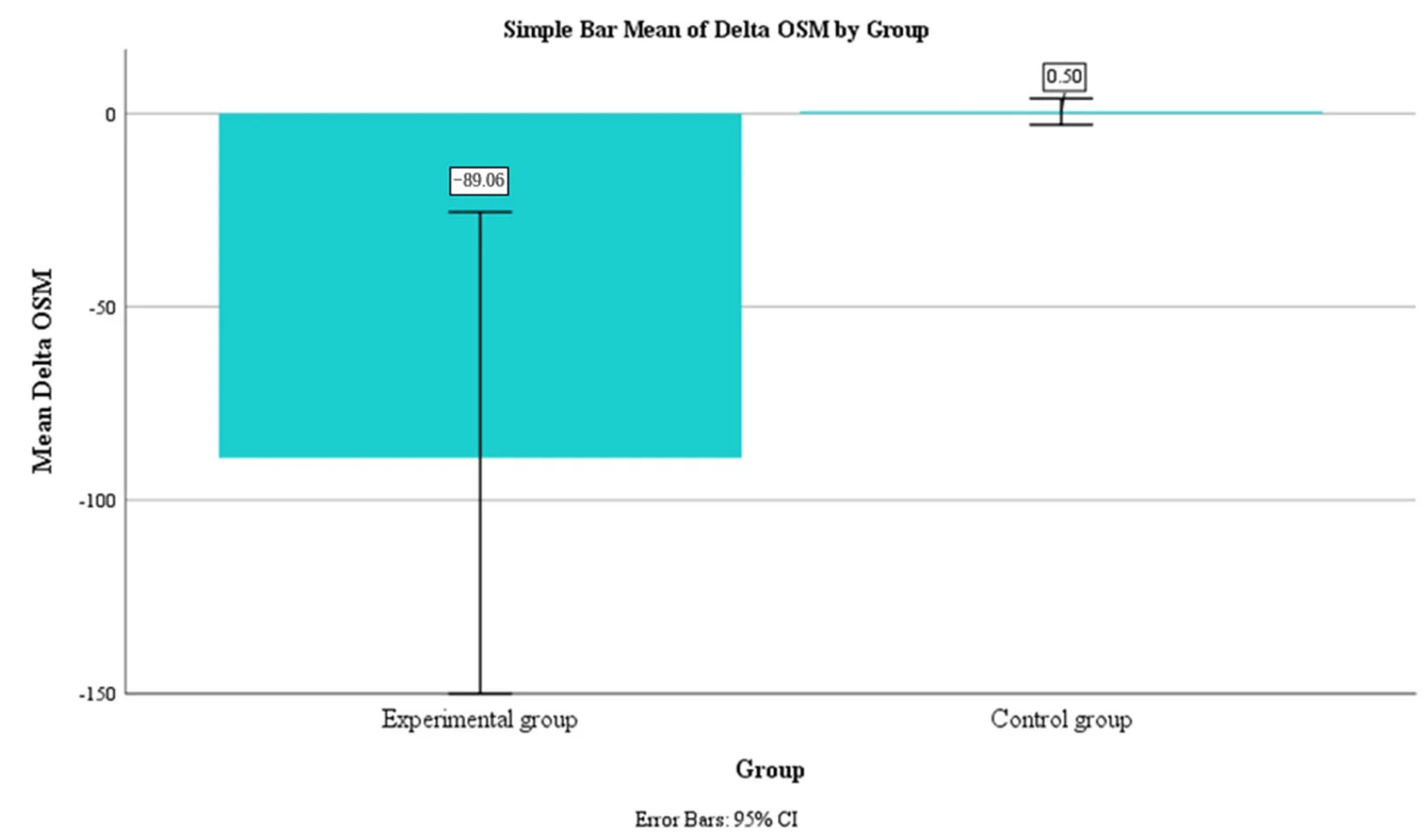
Change in ΔOSM between Visit 1 and Visit 2 in the study and control groups. Data are presented as mean ± 95% confidence interval.

The change in ΔOSM differed between groups. Using Welch’s *t*-test, the difference was not statistically significant when assuming equal variances, but the Welch-adjusted test indicated a significant group difference. Specifically, the study group showed a decrease in OSM levels (M = −89.06, SD = 320.38, SE = 32.04, *n* = 100), whereas the control group showed a slight increase (M = 0.50, SD = 9.12, SE = 1.67, *n* = 30), t(99.53) = −2.79, *p* = 0.006, mean difference = −89.56, 95% CI [−153.21, −25.91]—Figure 8.

### 2.5. Combined Biomarkers Assessment

A comparative ROC analysis using the DeLong test was performed to evaluate the predictive performance of OSM, FC, and the combined OSM + FC logistic model at Visit 1 and Visit 2 for identifying active disease at Visit 2 across three domains: clinical (pMS), endoscopic (MES), and histological (NHI).

#### 2.5.1. Clinical Activity (pMS)

**Figure 9:**
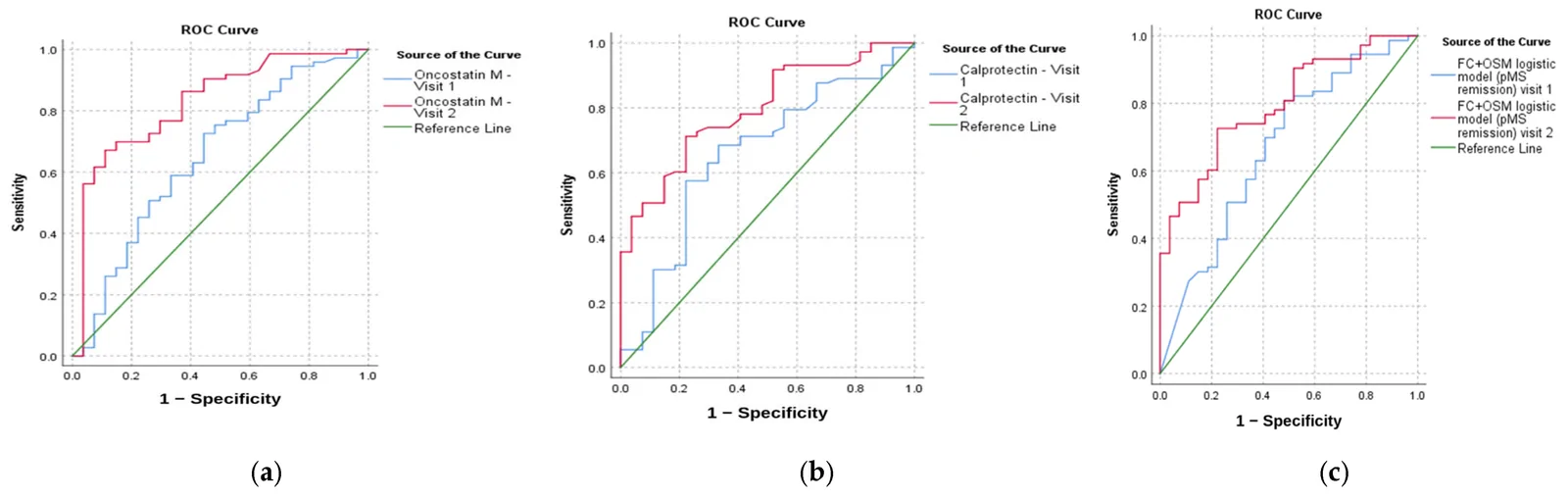
(**a**) Comparison of ROC curves for the diagnostic performance of OSM at Visit 1 versus Visit 2 in predicting active clinical disease based on the pMS. (**b**) Comparison of ROC curves for the diagnostic performance of FC at Visit 1 versus Visit 2 in predicting active clinical disease based on the pMS. (**c**) Comparison of ROC curves for the diagnostic performance of the FC + OSM logistic model (pMS remission) at Visit 1 versus Visit 2 in predicting active clinical disease based on the pMS.

Among the evaluated biomarkers, only OSM showed a statistically significant improvement in diagnostic performance over time. At Visit 2, OSM demonstrated good discriminative ability (AUC = 0.828, 95% CI: 0.744–0.912, *p* < 0.001), compared with a lower performance at Visit 1 (AUC = 0.650, 95% CI: 0.523–0.777, *p* = 0.011). The DeLong test confirmed that the increase in AUC of 0.178 was statistically significant (Z = −2.844, *p* = 0.004), indicating that OSM measured at Visit 2 was significantly more strongly associated with active clinical disease than OSM measured at Visit 1—Figure 9a. FC also showed improved diagnostic accuracy at Visit 2 (AUC = 0.797, 95% CI: 0.706–0.888) compared with Visit 1 (AUC = 0.659, 95% CI: 0.535–0.782). However, the observed increase in AUC of 0.138 did not reach statistical significance (Z = −1.885, *p* = 0.059)—Figure 9b. Similarly, the combined OSM + FC logistic model achieved a higher AUC at Visit 2 (0.796, 95% CI: 0.704–0.888) than at Visit 1 (0.667, 95% CI: 0.544–0.791), but this improvement was not statistically significant (ΔAUC = 0.129; Z = −1.588, *p* = 0.112)—Figure 9c.

Overall, these findings indicate that only OSM exhibited a significant enhancement in predictive performance between visits, whereas FC and the combined OSM + FC model showed numerical improvements that did not achieve statistical significance. Therefore, OSM measured at Visit 2 emerged as the marker with the strongest concurrent diagnostic association with active clinical disease in this analysis.

#### 2.5.2. Endoscopic Activity (MES)

**Figure 10:**
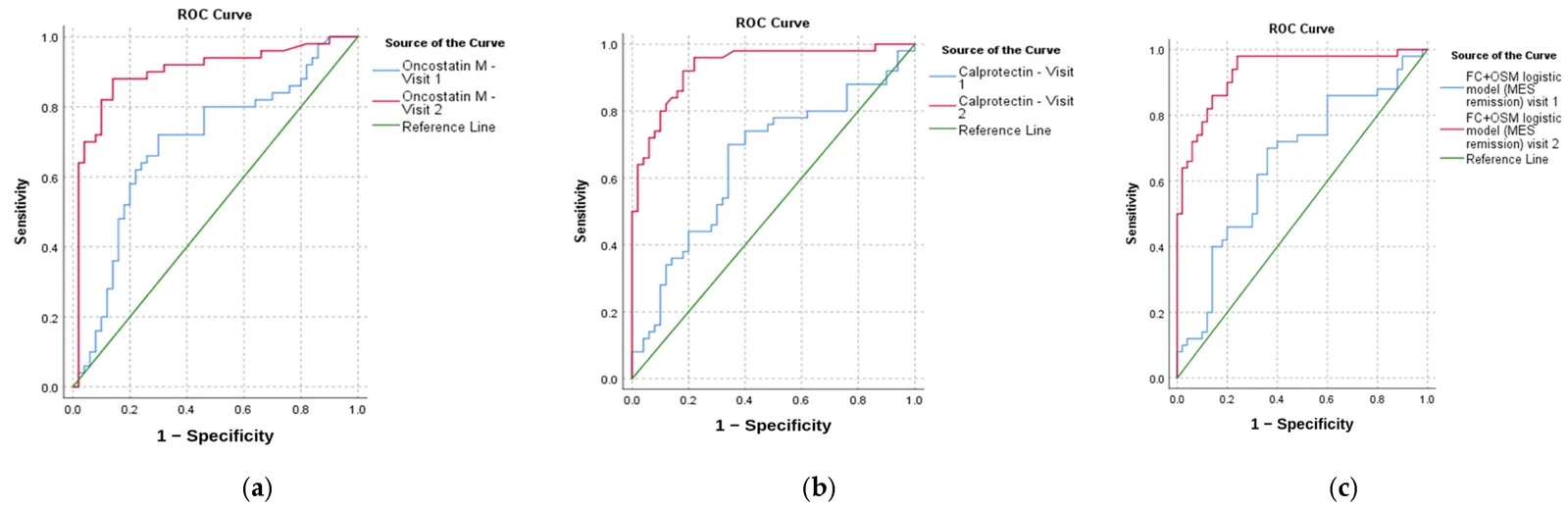
(**a**) Comparison of ROC curves for the diagnostic performance of OSM at Visit 1 versus Visit 2 in predicting active endoscopic disease based on the MES. (**b**) Comparison of ROC curves for the diagnostic performance of FC at Visit 1 versus Visit 2 in predicting active endoscopic disease based on the MES. (**c**) Comparison of ROC curves for the diagnostic performance of the combined score (FC + OSM) at Visit 1 versus Visit 2 in predicting active endoscopic disease based on MES.

All evaluated biomarkers demonstrated a statistically significant improvement in diagnostic performance between Visit 1 and Visit 2. OSM showed a substantial increase in diagnostic accuracy, with the AUC rising from 0.695 (95% CI: 0.588–0.801, *p* = 0.002) at Visit 1 to 0.897 (95% CI: 0.829–0.965, *p* < 0.001) at Visit 2. The DeLong test confirmed that this improvement was statistically significant (ΔAUC = 0.202; Z = −4.187; *p* < 0.001), indicating that OSM measured at Visit 2 was significantly more strongly associated with active endoscopic disease than OSM measured at baseline—Figure 10a. FC exhibited an even greater increase in discriminative performance, with the AUC improving from 0.652 (95% CI: 0.543–0.762, *p* = 0.008) at Visit 1 to 0.933 (95% CI: 0.885–0.982, *p* < 0.001) at Visit 2. This increase of 0.281 in AUC was highly significant (Z = −5.163; *p* < 0.001), demonstrating excellent diagnostic accuracy for detecting active endoscopic disease at Visit 2—Figure 10b.

Similarly, the combined OSM + FC logistic model showed a marked enhancement in diagnostic accuracy, with the AUC increasing from 0.657 (95% CI: 0.548–0.765, *p* = 0.006) at Visit 1 to 0.933 (95% CI: 0.884–0.982, *p* < 0.001) at Visit 2. The increase in AUC of 0.276 was statistically significant (Z = −5.150; *p* < 0.001), confirming that the combined model at Visit 2 provided substantially better discrimination of active endoscopic disease than the corresponding model at Visit 1—Figure 10c.

Overall, these findings demonstrate that all biomarkers achieved significantly higher predictive accuracy at Visit 2 compared with Visit 1 for identifying active endoscopic disease. Among the individual biomarkers, FC showed the greatest improvement in performance, while both FC and the combined OSM + FC model achieved the highest overall diagnostic accuracy (AUC = 0.933). These results highlight the superior value of follow-up biomarker assessment and support the use of both FC and OSM, particularly in combination, for monitoring endoscopic disease activity.

#### 2.5.3. Histological Activity (NHI)

**Figure 11:**
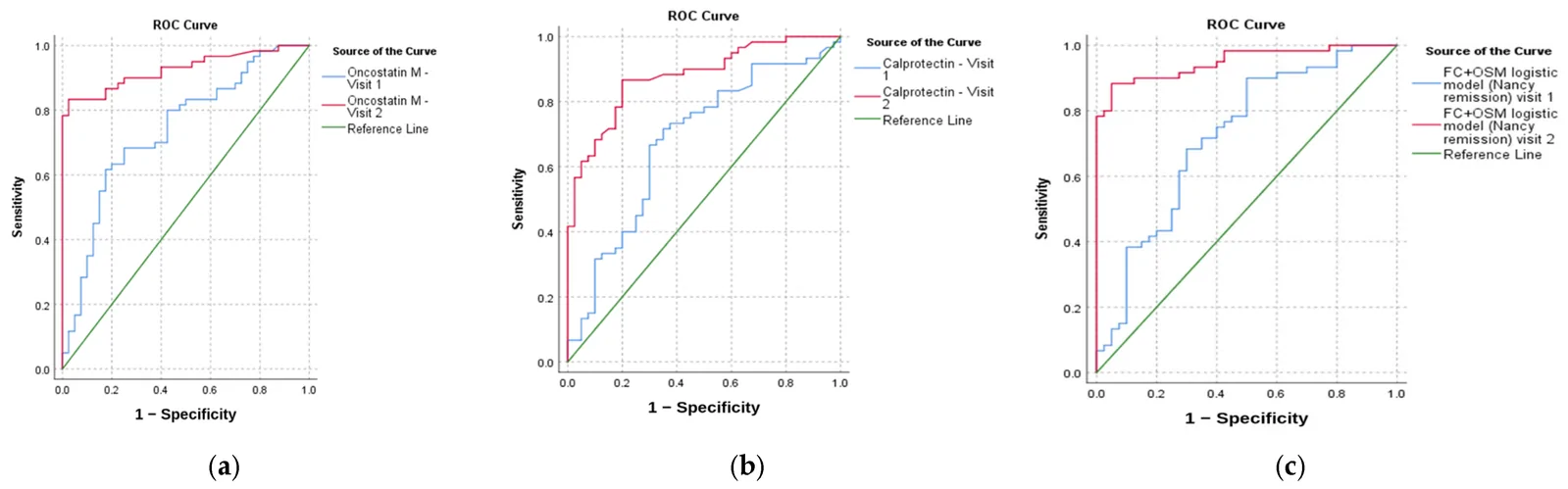
(**a**) Comparison of ROC curves for the diagnostic performance of OSM at Visit 1 versus Visit 2 in predicting active histological disease based on the NHI. (**b**) Comparison of ROC curves for the diagnostic performance of FC at Visit 1 versus Visit 2 in predicting active histological disease based on the NHI. (**c**) Comparison of ROC curves for the diagnostic performance of the combined score (FC + OSM) at Visit 1 versus Visit 2 in predicting active histological disease based on the NHI.

All evaluated biomarkers demonstrated a statistically significant improvement in diagnostic performance between Visit 1 and Visit 2. OSM showed a marked increase in diagnostic accuracy, with the AUC rising from 0.738 (95% CI: 0.638–0.838, *p* < 0.001) at Visit 1 to 0.927 (95% CI: 0.876–0.978, *p* < 0.001) at Visit 2. The DeLong test confirmed that this improvement was statistically significant (ΔAUC = 0.189; Z = −3.724; *p* < 0.001), indicating that OSM measured at Visit 2 was significantly more strongly associated with active histological disease than OSM measured at baseline—Figure 11a. FC also exhibited a significant enhancement in diagnostic performance, with the AUC increasing from 0.679 (95% CI: 0.570–0.788, *p* = 0.005) at Visit 1 to 0.878 (95% CI: 0.793–0.963, *p* < 0.001) at Visit 2. The observed increase in AUC of 0.199 was statistically significant (Z = −3.384; *p* = 0.001), demonstrating a markedly improved ability to discriminate active histological disease at follow-up—Figure 11b. The combined OSM + FC logistic model achieved the highest diagnostic accuracy among all evaluated biomarkers. Its AUC increased from 0.721 (95% CI: 0.616–0.826, *p* < 0.001) at Visit 1 to 0.950 (95% CI: 0.906–0.994, *p* < 0.001) at Visit 2. The improvement of 0.229 in AUC was statistically significant (Z = −3.960; *p* < 0.001), confirming that the combined model at Visit 2 provided superior discrimination of active histological disease compared with the same model at Visit 1—Figure 11c.

Overall, these findings demonstrate that all biomarkers showed significantly improved predictive accuracy at Visit 2 for identifying active histological disease according to the NHI. While both OSM and FC individually achieved excellent diagnostic performance at follow-up, the combined OSM + FC model yielded the highest overall accuracy (AUC = 0.950), highlighting the added value of integrating both biomarkers for the assessment and monitoring of histological disease activity.

To conclude, at baseline, OSM alone showed the highest discriminative ability for predicting active histological disease at follow-up (AUC = 0.738), outperforming both FC alone (AUC = 0.679) and the combined OSM + FC model (AUC = 0.721). These findings suggest that baseline OSM carries most of the diagnostic information relevant to later histological status, while the addition of FC does not provide a meaningful improvement in discriminative performance at that timepoint. At Visit 2, however, the combined model achieved the highest AUC (0.950), slightly exceeding OSM alone (0.927), suggesting a modest incremental value of combining biomarkers during follow-up assessment.

### 2.6. Plasma OSM Performance Relative to Complete Endoscopic Remission

Because complete endoscopic remission (MES 0) is associated with more favorable long-term outcomes than MES 1 and represents an increasingly stringent therapeutic target in current UC management, we performed an additional analysis evaluating plasma OSM specifically in relation to MES 0, distinct from the MES 0–1 vs. ≥2.

#### OSM Levels Across MES 0, MES 1, and MES ≥ 2

At Visit 1, the MES 0 subgroup was small (*n* = 4), limiting the reliability of a three-group comparison at baseline; this analysis is therefore reported for Visit 2 only, where subgroup sizes were adequate (MES 0, *n* = 23; MES 1, *n* = 27; MES ≥ 2, *n* = 50). At Visit 2, plasma OSM levels differed significantly across the three endoscopic subgroups (Kruskal–Wallis H = 51.22, *p* < 0.001), with a clear stepwise gradient from complete remission to active disease. Pairwise post hoc comparisons (Mann–Whitney U with Bonferroni correction for three comparisons) confirmed that OSM was significantly lower in patients with MES 0 than in those with MES 1 (*p* = 0.009), in addition to the expected significant differences between MES 0 and MES ≥ 2 (*p* < 0.001) and between MES 1 and MES ≥ 2 (*p* < 0.001). This indicates that plasma OSM distinguishes complete mucosal healing (MES 0) from milder residual endoscopic disease activity (MES 1), not only from more overt endoscopic inflammation.

Moreover, to directly evaluate plasma OSM as a marker of complete endoscopic remission, we performed ROC analysis using MES 0 as the reference standard, distinguishing it from MES ≥ 1 (any residual endoscopic activity), at Visit 2. Plasma OSM showed good discriminative accuracy (AUC = 0.878, 95% CI 0.812–0.945), comparable to FC (AUC = 0.824, 95% CI 0.739–0.908), with the combined OSM + FC logistic model achieving the highest accuracy (AUC = 0.899, 95% CI 0.838–0.960) (Table 5). The optimal OSM cutoff by the Youden index was 31.3 pg/mL (sensitivity 67.5%, specificity 100.0%).

DeLong’s test showed no statistically significant difference between the AUCs of OSM and FC alone (Z = 1.13, *p* = 0.261), indicating comparable individual discriminative performance for this stricter remission threshold. However, the combined OSM + FC model achieved a significantly higher AUC than FC alone (Z = 2.16, *p* = 0.031). This was corroborated by nested logistic regression model comparison: adding OSM to a FC-only model produced a highly significant improvement in model fit for distinguishing MES 0 from MES ≥ 1 (likelihood ratio χ^2^(1) = 18.10, *p* < 0.001).

Taken together, these results suggest that the incremental diagnostic value of plasma OSM over FC is threshold-dependent: OSM adds little beyond FC when the therapeutic target is the conventional MES 0–1 definition of endoscopic remission, but provides meaningful, statistically robust incremental value when the target is complete mucosal healing (MES 0). This distinction may be clinically relevant as treat-to-target strategies in UC increasingly move toward MES 0 as the preferred endoscopic endpoint.

## 3. Discussion

### 3.1. Summary of Main Findings

Our findings indicate that plasma OSM reflects disease activity in UC across clinical, endoscopic, and histological assessments, with correlations strengthening from Visit 1 to Visit 2 and OSM’s discriminative accuracy (AUC) improving over the course of follow-up, particularly for endoscopic (rising from 0.695 to 0.897) and histological (rising from 0.738 to 0.927) activity. This indicates that OSM’s concurrent association with mucosal and microscopic disease status is stronger when measured at the later follow-up visit than at baseline; however, since both AUCs are evaluated against activity measured at the same visit as the biomarker, this reflects diagnostic performance at each time point rather than evidence that OSM becomes a stronger prospective predictor over time. Longitudinal changes (ΔOSM) correlated moderately with ΔCalprotectin, ΔClinical Mayo, ΔMES, and ΔNANCY, but not with ΔCRP or ΔFibrinogen. In multivariable models, OSM’s dynamics were independently explained by histological (NANCY) and biochemical (FC) change, while in models assessing concurrent association with clinical or endoscopic status, FC was the dominant correlate, with OSM contributing little additional explanatory power. The exception was the model assessing concurrent association with histological status, where both ΔOSM and baseline OSM emerged as strong, independent correlates that complemented FC rather than being subsumed by it. Independently, baseline OSM alone also showed genuine prospective value over baseline FC for predicting subsequent histological status, after adjustment for baseline treatment exposure; this baseline-only association was not materially confounded by treatment escalation during follow-up.

Our results corroborate the landmark findings of West et al. [12], who demonstrated increased expression of OSM and its receptor (OSMR) in the inflamed intestinal mucosa of IBD patients relative to healthy controls, with levels correlating closely with histopathological disease severity. This provides biological plausibility for our findings that OSM correlated most strongly with histological disease activity (NHI): by acting directly on stromal cells to drive IL-6, ICAM-1, and chemokine production, OSM reflects tissue-level inflammation more directly than systemic markers do. A recent single-center study [13] reported effect sizes comparable to these results: plasma OSM was significantly associated with partial Mayo score (β = 0.471, *p* < 0.001), MES (β = 0.422, *p* < 0.001), NHI (β = 0.422, *p* < 0.001), and FC (β = 0.431, *p* < 0.001). Notably, OSM’s diagnostic accuracy for histological inflammation reached an AUC of 0.967, outperforming FC (AUC = 0.875), a finding that closely parallels our own Visit 2 histological AUC of 0.927. Together, these results reinforce that OSM’s strongest and most consistent diagnostic signal is for histological rather than clinical activity [13].

The lack of correlation between ΔOSM and ΔCRP is consistent with a growing body of literature that positions OSM as mechanistically and clinically distinct from classic acute-phase reactants. A recent review of UC biomarkers highlights plasma OSM as achieving diagnostic AUCs as high as 0.94 [14], placing it in the same tier as other emerging non-CRP biomarkers, precisely because CRP is known to underperform in a meaningful subset of UC patients whose inflammation is predominantly mucosal rather than systemic. This interpretation—that OSM’s weak correlation with CRP reflects biological specificity for local intestinal rather than systemic inflammation—is biologically plausible but not directly established by the present data, and should be regarded as a hypothesis warranting dedicated mechanistic or comparative validation.

Also, our findings of higher OSM in the study group versus controls, decreasing with treatment, align with West et al. [12], which linked elevated pre-treatment OSM to anti-TNF non-response in over 200 IBD patients. This has since been extended by a retrospective cohort study showing that plasma OSM concentrations measured prior to anti-TNF exposure were associated with clinical remission at 1 year in both UC and CD [15]. Although our study did not specifically evaluate anti-TNF response, the direction of our findings—OSM tracking inflammatory burden and its diagnostic performance improving with treatment-related change—is consistent with this literature and may serve as an important focus for future investigation.

OSM vs. FC as complementary, not competing, markers. Our logistic regression findings—FC dominating for clinical/endoscopic remission, but OSM and FC each independently significant for histological remission—parallel comparative biomarker literature. Cao et al. [16] similarly positioned plasma OSM as a surrogate biomarker for IBD activity and infliximab response when evaluated against standard markers, reporting that patients without mucosal healing had higher plasma OSM levels than those with mucosal healing (AUC = 0.843), and that OSM levels were elevated in clinical non-responders compared to responders (AUC = 0.898). These findings suggest that OSM may provide complementary diagnostic information alongside FC. In our cohort, combining OSM with FC yielded a modest but consistent improvement in AUC compared with FC alone, with performance (AUC 0.84–0.93) comparable to that reported by Cao et al. [16].

Importantly, the incremental value of OSM over FC was not uniform across definitions of endoscopic remission. When endoscopic remission was defined conventionally as MES 0–1 versus ≥2, OSM added no significant diagnostic information beyond FC (Section 2.3.4). However, when we applied the stricter definition of complete endoscopic remission (MES 0) versus any residual activity (MES ≥ 1), OSM levels differed significantly not only between active and inactive disease but also between MES 0 and MES 1 specifically (*p* = 0.009), and adding OSM to FC produced a significant improvement in both model fit (likelihood ratio χ^2^(1) = 18.10, *p* < 0.001) and discriminative accuracy (DeLong Z = 2.16, *p* = 0.031) (Section 2.6). This threshold-dependence suggests that OSM’s complementary value over FC is most apparent when the clinical question is whether mucosal healing is complete, rather than whether disease activity is merely mild. Given the growing emphasis on MES 0 as a treat-to-target endpoint in UC [4], this represents a clinically relevant and, to our knowledge, previously unreported nuance in the comparative performance of these two biomarkers.

Regarding disease extent, we identified a significant, though weak, negative Spearman correlation between ΔOSM and disease extent (E1–E3) (ρ = −0.226, *p* = 0.024)—an association not previously reported in the OSM literature, which has largely focused on activity rather than extent. Given the modest effect size and single-center design, this potentially novel observation should be interpreted cautiously and validated in larger, independent cohorts.

### 3.2. Strengths and Limitations

This study offers several strengths relative to the existing OSM literature. Unlike most published OSM studies, which are cross-sectional single-time point analyses, this study assessed OSM against clinical, endoscopic, and histological endpoints at two visits, enabling both static correlation and dynamic (Δ) analysis—a more clinically meaningful test of whether OSM change actually parallels disease-course change. This was paired with use of the NHI as the histological reference standard, providing a validated, reproducible ground truth rather than relying on endoscopy or symptom-based scores alone. Comparative ROC analysis using the DeLong test across time points—largely absent from prior OSM literature—further allowed us to directly test whether OSM’s diagnostic value at follow-up differs statistically from baseline, rather than simply reporting two separate AUCs, and this rigor was supported by consistent multicollinearity diagnostics (VIF) across all regression models. Beyond statistics, our findings show biological coherence: the dissociation between OSM/FC (tissue-driven markers) and CRP/fibrinogen (systemic acute-phase reactants) is consistent with OSM’s proposed mechanism as a stromal/mucosal cytokine rather than a hepatocyte-derived acute-phase protein. Finally, to our knowledge, this is among the first studies to report a significant, though weak, negative Spearman correlation between ΔOSM and disease extent (E1–E3) (ρ = −0.226, *p* = 0.024)—an association not previously described in the OSM literature, which has predominantly focused on activity rather than extent. Given the modest effect size, this should be considered hypothesis-generating and warrants validation in larger, independent cohorts.

This study also has several limitations. The single-center design and modest sample size (*n* = 100 patients, 30 controls) limit generalizability and reduce power for subgroup analyses, raising the possibility that borderline associations, such as the correlation with disease extent (*p* = 0.024), reflect sampling variability rather than a true population effect. The two-visit design captures change over a single interval, precluding causal inference and limiting insight into OSM trajectories across multiple relapse-remission cycles; heterogeneous treatment exposures between visits (5-ASA, corticosteroids, biologics, advanced therapy) represent a further potential confounder for ΔOSM that was not fully controlled for. Although data on baseline and follow-up use of therapies were collected for all patients at both visits, a detailed treatment-stratified analysis was not performed in this manuscript and is planned as a separate, dedicated report. Consequently, we cannot exclude the possibility that differences in therapeutic exposure between visits contributed to the observed improvements in clinical, endoscopic, and biomarker activity, and this should be considered when interpreting the magnitude of change reported here. In addition, for a subset of patients, treatment was modified in the interval between biomarker sampling and endoscopic/histological assessment (maximum 1 month, mean approximately 1 week); because biomarker levels were measured before any such change while the endoscopic and histological outcome reflects status afterward, this introduces a potential source of misalignment between predictor and outcome timing that could not be excluded in the present analysis and should be considered when interpreting the strength of the reported associations. Given that Visit 1 and Visit 2 samples were analyzed on different ELISA plates, we cannot exclude the possibility that plate-to-plate or inter-assay variability contributed to the observed longitudinal change in OSM; this is acknowledged as a limitation of the present analysis. Moreover, detailed demographic characterization of the healthy control group (age, sex, BMI, smoking status) was not implemented, since controls were recruited to establish reference OSM values rather than as a matched comparator group.

As all ROC/AUC estimates and regression coefficients were derived and evaluated within the same sample, without external validation, diagnostic performance may be inflated relative to an independent replication cohort. Additionally, OSM was measured using a single ELISA platform; since assay methodology and absolute values are not standardized across studies, direct numeric comparison with other cohorts is limited. Disease extent was assessed using a coarse ordinal proxy (E1–E3, Montreal classification), and more granular endoscopic scoring might reveal a stronger relationship with ΔOSM. Finally, we did not directly assess anti-TNF or biologic response, despite this being the domain with the strongest prior evidence for OSM [12]—a clear direction for future work with this cohort.

### 3.3. Economic and Practical Implications

Although we did not perform a formal cost-effectiveness analysis, several practical implications merit discussion. Endoscopy with histological sampling remains the reference standard for assessing disease activity in UC but is invasive, resource-intensive, and poorly suited to frequent monitoring. Non-invasive plasma biomarkers such as OSM and FC could reduce reliance on surveillance colonoscopies, particularly in patients with stable or improving trends. As OSM showed the strongest correlation with histological activity and combined OSM + FC models modestly outperformed FC alone, incorporating OSM into monitoring algorithms may, if validated in future prospective studies, help reduce reliance on surveillance endoscopy; however, the present study does not directly demonstrate that OSM can replace endoscopy or independently guide clinical decision-making, and this remains a hypothesis for future testing rather than an established application.

Overall, these findings position OSM as a biologically coherent and diagnostically promising biomarker in UC, warranting further validation in larger, independent, and multicenter cohorts before its role in routine clinical practice can be firmly established.

## 4. Materials and Methods

### 4.1. Inclusion and Exclusion Criteria Ethical Approval

Of 135 patients screened for eligibility, 20 were excluded (associated infections, rheumatologic or other systemic immune-mediated disease, or other exclusion criteria listed above), leaving 115 enrolled patients. Of these, 15 did not complete the study (transfer of care to another clinic or voluntary withdrawal) and were excluded from the final analysis. The remaining 100 patients completed both study visits with complete paired data across all core clinical, endoscopic, histological, and biomarker variables (OSM, FC, pMS, MES, NHI); no missing values were present in these fields. Therefore, a total of 100 eligible participants (≥18 years), both male and female, had a confirmed diagnosis of UC based on clinical, endoscopic, and histological criteria in accordance with the ECCO guidelines [17]. Demographic information, clinical assessments, and medical histories were recorded for all patients. All patients underwent extensive clinical and paraclinical evaluation. For all included patients, we collected data on the diagnosis date, disease extension, clinical assessment, endoscopic activity, and histological activity. Plasma samples were collected from all UC patients, whether in remission or experiencing active disease. Exclusion criteria included indeterminate colitis, bacterial superinfection, colorectal cancer or prior malignancy, and other systemic immune-mediated diseases (e.g., rheumatoid arthritis).

The present study was approved by the Ethics Committees of Grigore T. Popa University of Medicine and Pharmacy Iasi, Romania, and the “Saint Spiridon” County Clinical Emergency Hospital Iasi, Romania. The study was conducted in accordance with the ethical guidelines outlined in the Declaration of Helsinki. All participants provided written informed consent.

### 4.2. Patient Recruitment and Sample Collection

Each patient underwent two main study visits: one at baseline (study enrollment, Visit 1) and one at 12 months (Visit 2). At each visit, blood was drawn for plasma OSM, FC, CRP, and fibrinogen, and clinical activity (pMS) was assessed; endoscopy with biopsy (for MES and NHI) was performed within a mean interval of approximately 1 week of blood sampling at each visit, accounting for the sampling-to-endoscopy interval. Treatment status was recorded at each visit. Blood for OSM measurement was collected in K2-EDTA tubes, processed within 1–2 h, and plasma aliquots were extracted via centrifugation (1000× *g*, 20 min, 18 °C) and stored at −80 °C for periods ranging from 3 to 16 months, depending on the collection date, to avoid freeze–thaw cycles until batch analysis. The same collection and processing protocol was used for all patients at both visits and for healthy controls. This storage duration is well within the established stability window for cytokines, as prior studies have demonstrated that serum and plasma cytokines remain stable for at least 2 years when kept at −80 °C [18]. A commercial enzyme-linked immunosorbent assay (ELISA) kit—Human OSM (oncostatin M) ELISA Kit (E-EL-H2247, 96T; Elabscience Biotechnology Inc., Houston, TX, USA)—was used for the colorimetric detection and quantification of human OSM as per the manufacturer’s protocol, with a sensitivity of 9.38 pg/mL. OSM plasma levels were measured in pg/mL. Samples underwent a single freeze–thaw cycle prior to analysis and were not run in duplicate. OSM was measured across 3 ELISA plates; Visit 1 and Visit 2 samples from the same patient were analyzed on separate plates. Laboratory personnel performing the OSM assay were blinded to patients’ clinical, endoscopic, and histological disease activity.

At the main visits, the following tests were performed in our department: complete blood count, CRP, and fibrinogen. FC was determined by ELISA test and was collected before bowel preparation. Clinical, endoscopic, and histologic activity were assessed as follows: disease extension using the Montreal classification: E1 proctitis, E2 left side colitis, E3 pancolitis; clinical activity using the partial Mayo score (pMS—total score 9): remission ≤ 2; endoscopic activity using the Mayo Endoscopic Score (MES 0–3) according to vascular pattern, bleeding, and ulcers: remission 0–1, mild 2, moderate-to-severe 3; histologic activity using the Nancy Histological Index (NHI 0–4): remission 0–1, active disease ≥ 2. Endoscopy and biopsy were performed within a maximum of 1 month from the time of sample collection (mean interval approximately 1 week). Histological assessment was performed by a single pathologist, blinded to clinical, endoscopic, and biomarker results. At each visit, 2–4 targeted biopsies were obtained under narrow-band imaging (NBI) guidance from the most inflamed mucosal segment; where biopsies yielded differing Nancy Histological Index scores, the highest (most severe) score was used as the final NHI for that visit. In patients with severe active disease, colonoscopy was occasionally incomplete, and biopsies were obtained from the segment(s) reached during the procedure.

### 4.3. Study Design and Data Analysis

This was a single-center, prospective study including 100 patients diagnosed with UC and 30 healthy controls. Patient data and blood samples were collected and analyzed from April 2024 to March 2026. Study participants were evaluated at two time points (Visit 1—baseline and Visit 2—at 12 months) to assess clinical, endoscopic, histological, and biochemical disease activity, as well as plasma OSM and FC levels. Clinical activity was assessed using the partial Mayo score (pMS), endoscopic activity using the Mayo Endoscopic Score (MES), and histological activity using the Nancy Histological Index (NHI). Disease extent was classified according to the Montreal classification (E1–E3). Disease severity was additionally categorized using the Truelove and Witts classification. Control subjects underwent also two OSM measurements for comparison. In each logistic regression model, the dependent variable was coded 1 = active disease and 0 = remission (clinical: pMS > 2 vs. ≤2; endoscopic: MES ≥ 2 vs. 0–1, with MES ≥ 1 vs. 0 evaluated separately in Section 2.6; histological: NANCY ≥ 2 vs. 0–1).

IBM SPSS Statistics (v26.0) software was used for the statistical processing. Descriptive statistics were calculated for all study variables, with continuous variables expressed as mean ± standard deviation (SD) and categorical variables as frequencies and percentages. Associations between continuous variables (OSM, FC, pMS, CRP, fibrinogen) were assessed using Pearson correlation coefficients, while the relationship between ΔOSM and the ordinal variable disease extent (E1–E3) was assessed using Spearman’s rank correlation. Correlation analyses were performed separately at Visit 1, Visit 2, and for delta (Δ) values between visits. Between-group comparisons of OSM levels (study vs. control group) were performed using independent-samples *t*-tests; where Levene’s test indicated unequal variances, Welch’s correction was applied. Multiple linear regression was used to identify predictors of ΔOSM, incorporating ΔCalprotectin, ΔNANCY, and Nancy remission status at Visit 2 as covariates. Binary logistic regression models, adjusted for baseline (Visit 1) values, were used to evaluate the predictive value of ΔOSM and ΔCalprotectin for active clinical, endoscopic, and histological disease at Visit 2. Multicollinearity was assessed for all regression models using Variance Inflation Factor (VIF) values.

Diagnostic performance of OSM, FC, and the combined OSM + FC logistic model was evaluated using Receiver Operating Characteristic (ROC) curve analysis, with the DeLong test used to statistically compare AUCs between Visit 1 and Visit 2 for each biomarker and disease assessments (clinical, endoscopic, histological). To evaluate plasma OSM specifically in relation to complete endoscopic remission, OSM levels were additionally compared across three MES subgroups (0, 1, and ≥2) using the Kruskal–Wallis test with Bonferroni-corrected pairwise Mann–Whitney comparisons, and ROC/DeLong analysis was performed using MES 0 as the reference standard for remission versus MES ≥ 1. Internal validation of the primary histological model was performed via bootstrap resampling (500 iterations) with optimism correction.

A *p*-value < 0.05 was considered statistically significant for all analyses.

## 5. Conclusions

Plasma OSM appears to be a promising biomarker for evaluating disease activity in UC across clinical, endoscopic, and histological measures. Its correlation with histological activity was consistently the strongest across both visits, and it emerged as an independent predictor of histological remission alongside FC—a pattern that aligns with OSM’s proposed mechanism of acting directly on mucosal stromal cells to drive local inflammation, in contrast to CRP, which primarily reflects the systemic acute-phase response. The persistently weak correlation between OSM and CRP throughout the study further suggests that OSM provides distinct, non-overlapping information rather than simply mirroring systemic inflammation. Consequently, OSM may serve as a complementary biomarker, particularly for identifying tissue-level inflammatory activity that is not fully reflected by conventional clinical assessments or routine biochemical markers. This tissue-specific inflammatory signature may explain its superior association with histological disease activity and its potential to identify ongoing mucosal inflammation that is not fully reflected by conventional clinical or biochemical assessments.

Taken together, these findings support the incorporation of plasma OSM as a complementary biomarker to FC for monitoring mucosal and histological healing in UC, with this complementary value being most pronounced when the therapeutic target is complete endoscopic remission (MES 0) rather than the broader MES 0–1 definition. This domain- and threshold-dependence, rather than any single AUC or odds ratio, is the central and most clinically actionable finding of this study: OSM’s complementary value appears concentrated in exactly the settings—deep mucosal and histological healing.

Taken together, these findings support consideration of plasma OSM as a complementary, non-invasive marker to FC for monitoring mucosal and histological healing in UC, with its greatest potential utility where treat-to-target strategies increasingly demand confirmation of deep, rather than merely clinical, remission. Nevertheless, prospective multicenter studies with larger patient populations and extended follow-up are required to validate these observations and to further define the role of OSM within these strategies.

## Figures and Tables

**Table 1:** Baseline demographic, clinical, endoscopic, and laboratory characteristics of the study population at Visit 1 and Visit 2.

Characteristic			Category/Statistic	Value
Demographics				
Age (years)			Mean ± SD	41.96 ± 14.39
Gender			Male/Female, *n* (%)	62 (62.0%)/38 (38.0%)
Height (cm)			Mean ± SD	170.10 ± 7.63
Weight (kg)			Mean ± SD	74.52 ± 15.04
BMI (kg/m^2^)			Mean ± SD	25.24 ± 4.21
Lifestyle & Comorbidities				
Smoking Status			Active Smoker, *n* (%)	10 (10.0%)
Alcohol Consumption			Yes, *n* (%)	2 (2.0%)
Extraintestinal manifestations			Yes, *n* (%)	29 (29.0%)
Disease Phenotype				
Disease Extent (Montreal)			Proctitis (E1), *n* (%)	6 (6.0%)
			Left-sided (E2), *n* (%)	39 (39.0%)
			Pancolitis (E3), *n* (%)	55 (55.0%)
Clinical & Endoscopic Scores			Visit 1	Visit 2
Clinical MAYO Score			4.07 ± 1.82	3.28 ± 2.21
Endoscopic MAYO Score			1.86 ± 0.84	1.40 ± 0.98
NANCY Score			2.04 ± 1.19	1.92 ± 1.26
TrueLove & Witts (Mild/Mod/Sev)			31%/47%/22%	58%/34%/8%
Laboratory Biomarkers			Visit 1	Visit 2
Fibrinogen			374.84 ± 79.93	357.02 ± 61.63
CRP			1.32 ± 2.33	0.71 ± 0.54
Variable	Mean ± SD	Median	IQR (Q1–Q3)	Skewness
OSM Visit 1	222.36 ± 398.43	64.21	21.44–219.16	3.14
OSM Visit 2	133.30 ± 242.48	32.01	13.88–146.10	3.59
FC Visit 1	692.77 ± 1162.38	250.50	91.75–744.00	3.42
FC Visit 2	417.97 ± 637.72	132.50	55.25–471.50	2.65

**Table 2:** Correlations between ΔOncostatin M and clinical, biochemical, endoscopic, histologic, and categorical variables (*n* = 100).

Variable	Type	r/ρ	*p*-Value
ΔCalprotectin	Pearson	0.446	<0.001
ΔClinical Mayo score	Pearson	0.452	<0.001
ΔMES	Pearson	0.446	<0.001
ΔNANCY	Pearson	0.461	<0.001
ΔFibrinogen	Pearson	0.127	0.208
ΔCRP	Pearson	0.096	0.343
Age	Pearson	0.160	0.113
Body mass index	Pearson	0.133	0.188
Disease extent (E1–E3)	Spearman	−0.226	0.024

**Table 3:** Nested model comparison of FC alone versus FC + OSM for predicting active disease at Visit 2, across clinical, endoscopic, and histological domains. Likelihood ratio (LR) tests compare model fit; DeLong tests compare AUC. Significant improvement with the addition of OSM was observed only for histological remission.

Outcome	FC-Alone AUC	FC + OSM AUC	LR χ^2^ (df = 2)	LR *p*	DeLong Z	DeLong *p*
Clinical remission	0.793	0.788	0.69	0.710	0.38	0.707
Endoscopic remission	0.936	0.926	3.51	0.173	0.88	0.377
Histological remission	0.882	0.949	35.35	<0.001	−2.14	0.033

**Table 4:** Treatment exposure changes over the follow-up period.

Treatment Category	Visit 1	Visit 2
5-ASA	59%	48%
Corticosteroid	24%	2%
Immunomodulator (AZA/MTX)	11%	12%
Advanced therapy (biologic/small molecule)	62%	83%
Any treatment change between visits	—	41/100
Escalated to advanced therapy	—	21/100

**Table 5:** ROC performance of OSM, FC, and the combined OSM + FC model for discriminating MES 0 from MES ≥ 1 (Visit 2, *n* = 100).

Biomarker	AUC (95% CI)	Optimal Cutoff	Sensitivity	Specificity
Plasma OSM	0.878 (0.812–0.945)	31.3 pg/mL	67.5%	100.0%
FC	0.824 (0.739–0.908)	103 µg/g	74.0%	87.0%
OSM + FC (combined)	0.899 (0.838–0.960)	—	—	—

## Data Availability

Data are available from the corresponding author on reasonable request and with the permission of the institution.
